# Genome-Wide Analysis of microRNA and mRNA Expression in Colorectal Intramucosal Neoplasia and Colorectal Cancer With a Microsatellite-Stable Phenotype Based on Adenoma-Carcinoma Sequences

**DOI:** 10.3389/fonc.2022.831100

**Published:** 2022-07-07

**Authors:** Tamotsu Sugai, Mitsumasa Osakabe, Takeshi Niinuma, Ryo Sugimoto, Makoto Eizuka, Yoshihito Tanaka, Naoki Yanagawa, Koki Otsuka, Akira Sasaki, Takayuki Matsumoto, Hiromu Suzuki

**Affiliations:** ^1^ Department of Molecular Diagnostic Pathology, School of Medicine, Iwate Medical University, Shiwagun’yahabachou, Japan; ^2^ Department of Molecular Biology, Sapporo Medical University, School of Medicine, Sapporo, Japan; ^3^ Division of Gastroenterology, Department of Internal Medicine, Shiwagun’yahabachou, Japan; ^4^ Department of Surgery, School of Medicine, Iwate Medical University, Shiwagun’yahabachou, Japan

**Keywords:** adenoma, adenoma-carcinoma sequence, array-based analysis, microRNA, messenger RNA

## Abstract

**Background:**

Although MicroRNAs (miRNAs) play important roles in various biological processes, the biological functions of miRNAs are achieved through mRNAs. The aim of this study is to identify dysregulated miRNA/mRNA expression patterns in colorectal tumors.

**Methods:**

We examined 42 colorectal tumors [15 adenomas, 8 intramucosal cancers (IMCs), and 19 invasive colorectal cancers (CRCs)] with the microsatellite stable (MSS) phenotype (first cohort). The first cohort was used for genome-wide miRNA and mRNA expression arrays, whereas the second cohort (37 colorectal neoplasias) was used for validation analyses. Finally, we used 15 cases of “adenoma in/with carcinoma” to identify network patterns of miRNAs/mRNAs that were directly associated with neoplastic progression. In addition, simple regression analysis for array-based and RT-PCR analyses was performed to select candidate miRNA–mRNA pairs. Transfection of miRNA mimics was also performed to confirm whether target mRNA expression is affected by specific miRNAs.

**Results:**

Specific paired miRNA/mRNA networks, including hsa-miR-34a-5p/SLC12A2, hsa-miR-15b-5p/SLC12A2, hsa-miR-195-5p/SLC12A2, hsa-miRNA-502-3p/OLFM4, hsa-miRNA-6807-5p/ZG16, and hsa-miRNA 3064-5p/SH3BGRL3, were identified in samples of adenoma, IMC, and CRC with the MSS phenotype. In adenomatous lesions obtained from the same tumor with a carcinomatous lesion, we identified pairs of miRNA-130a-3p/HSPA8 and miRNA-22-3p/RP53 that were linked to multiple pathways. On the other hand, 2 pairs of miRNA/mRNA (miRNA-660-5p and miRNA-664a-5p/APP) were found in isolated carcinomatous glands. Ectopic expression of miRNA 3064-5p suppressed SH3BGRL3 expression.

**Conclusions:**

We found that networks based on specific pairs of miRNAs/mRNAs contribute to progression from adenomatous and carcinomatous lesions. Our results provide insights into the molecular tumorigenesis of colorectal tumors.

## Introduction

Fearon and Vogelstein proposed a novel hypothesis, designated the adenoma-carcinoma sequence of the colorectum, that describes transformation of normal colorectal epithelium to an adenoma and finally to an invasive and metastatic cancer (1). Based on this hypothesis, chromosomal instability, microsatellite instability, and CpG island methylator phenotype pathways contribute to genetic alterations occurring in colorectal cancer (2–4). Moreover, the adenoma-carcinoma progression is characterized by a chromosomal instability pathway that activates proto-oncogenes (*KRAS*) and inactivates at least 3 tumor suppression genes, including loss of *APC*, *TP53*, and *DPC4* (5–7). Mutation of the *PIK3CA* gene has also been recently described (7).

MicroRNAs (miRNAs) are small, noncoding RNAs that cannot be translated into proteins (8, 9). Notably, miRNAs have many essential roles in multiple biological processes. (8, 9). For example, miRNAs have been shown to modulate the expression of greater than 30% of genes in the human genome (10, 11). Moreover, dysregulation of miRNA expression has been shown to be related to various biological functions, such as cell differentiation, apoptosis, and carcinogenesis (12, 13). Many studies have shown that miRNAs play central roles in the occurrence and development of a variety of cancers, including lung cancer (14), breast cancer (15), and colon cancer (9). Most of the effects of miRNAs on biological functions occur *via* modulation of messenger RNAs (mRNAs), which are transcribed into proteins (16). Thus, the link between dysregulated expression of miRNA and altered expression of mRNA is important for human carcinogenesis and should be carefully evaluated.

Here, we aimed to evaluate dysregulated mRNA–miRNA pairs contributing to colorectal carcinogenesis. To this end, we carried out integrative analyses of the interactions between miRNAs and target genes by applying high-throughput molecular profiling of isolated colorectal adenoma and cancerous samples, including colorectal intramucosal cancer (IMC) and invasive CRC. We also validated additional isolated tumor samples. In addition, we used samples of intramucosal neoplasia (intramucosal adenocarcinoma accompanied by an adenoma component, equivalent to pT1 carcinoma) to identify molecular alterations of direct progression from adenomatous to cancerous lesions within the same tumor.

## Methods

### Patients

In total, 42 patients were enrolled as the first cohort in this study; these patients included 15 cases of colorectal adenoma (both tubular and tubulovillous adenomas), 8 cases of IMC, and 19 cases of CRC (with invasion beyond the muscular layer without metastasis). Adenoma and IMC were diagnosed histologically based on the World Health Organization (WHO; 2019) criteria, with some modifications (7). The adenomas evaluated in this study consisted of both low-grade adenomas (LGAs) and high-grade adenomas (HGAs). The clinicopathological findings for the patients were described based on the General Rules for Management of the Japanese Colorectal Cancer Association (17). Furthermore, we performed a validation analysis using 37 colorectal tumors (15 adenomas, 8 IMCs, and 14 invasive CRCs) as the second cohort. **Table 1-a** shows the clinicopathological findings of the patients. All samples, including those from cohorts 1 and 2, were obtained from Iwate Medical University from 2018 to 2020. Therefore, samples from cohorts 1 and 2 were independently collected from the same hospital (Iwate Medical University).

**Table 1-A:** Clinicopathological findings of the colorectal lesions.

	Cohort 1 (microarray) (%)		Cohort 2 (validation test) (%)	
Sex				
Male	29	(69)	19	(51.4)
Female	13	(31)	18	(48.6)
Age, median (range)	67	(43-81)	67	(46-85)
Location				
C/A/T/D/S/R	2/8/6/3/11/12		4/10/1/1/8/13	
Histological type				
Conventional adenoma	15	(35.7)	15	(40.5)
Intramucosal cancer	8	(19)	8	(21.6)
Colorectal cancer with an MSS phenotype	19	(45.2)	14	(37.8)
MDA	18	(94.7)	13	(92.9)
MUC	1	(5.3)	1	(7.1)
Stage				
I	3	(15.8)	4	(28.6)
II	6	(31.6)	5	(35.7)
III	7	(36.8)	4	(28.6)
IV	3	(15.8)	1	(7.1)

C, cecum; A, ascending colon; T, transverse colon; D, descending colon; S, sigmoid colon; R, rectum; MSS, microsatellite stable; MDA, moderately differentiated adenocarcinoma; MUC, mucinous carcinoma.

We examined 15 cases of “adenoma in/with carcinoma” (within the same tumor) to identify network patterns of miRNAs/mRNAs that were directly associated with neoplastic progression. Histological diagnosis of the second cohort was made using the same criteria as the first cohort. The detailed clinicopathological findings which were described in accordance with the General Rules for Management of the Japanese Colorectal Cancer Association are summarized in **Table 1-B**. Overall, two types including lesions separately examined in each lesion (adenomas, IMCs, and CRCs with the microsatellite stable (MSS) phenotype; type A) and two lesions consisted with adenomatous and carcinomatous lesions within the same tumor (type B) were investigated in the present study.

**Table 1-B:** Clinicopathological findings of the colonic adenocarcinomas in/with adenoma.

	Clinicopathological findings (%)	
Total	15	
Sex		
Male	7	(46.7)
Female	8	(53.3)
Age, median (range) (years)	71 (53-83)	
Size, median (range) (mm)	40 (23-79)	
Location		
Right	9	(60)
Left	6	(40)
Macroscopic type		
Elevated	15	(100)
Depressed	0	(0)
Adenoma component		
Histological subtype		
Tubular adenoma	8	(53.3)
Tubulovillous adenoma	7	(46.7)
Histological grade		
Low grade	7	(46.7)
High grade	8	(53.3)
Presence of *KRAS* mutation	7	(46.7)
Carcinoma component		
Histological subtype		
Well differentiated	12	(80)
Moderately differentiated	2	(13.3)
Papillary	1	(6.7)
Depth of cancer invasion		
Mucosa	12	(80)
Submucosa	3	(20)
Presence of lymphatic invasion	0	(0)
Presence of venous invasion	0	(0)
Presence of *KRAS* mutation	9	(60)

This study was approved by the local ethics committee of Iwate Medical University (approval number MH2020-066), and all patients provided informed consent.

### Crypt Isolation Method

**Figure 1:**
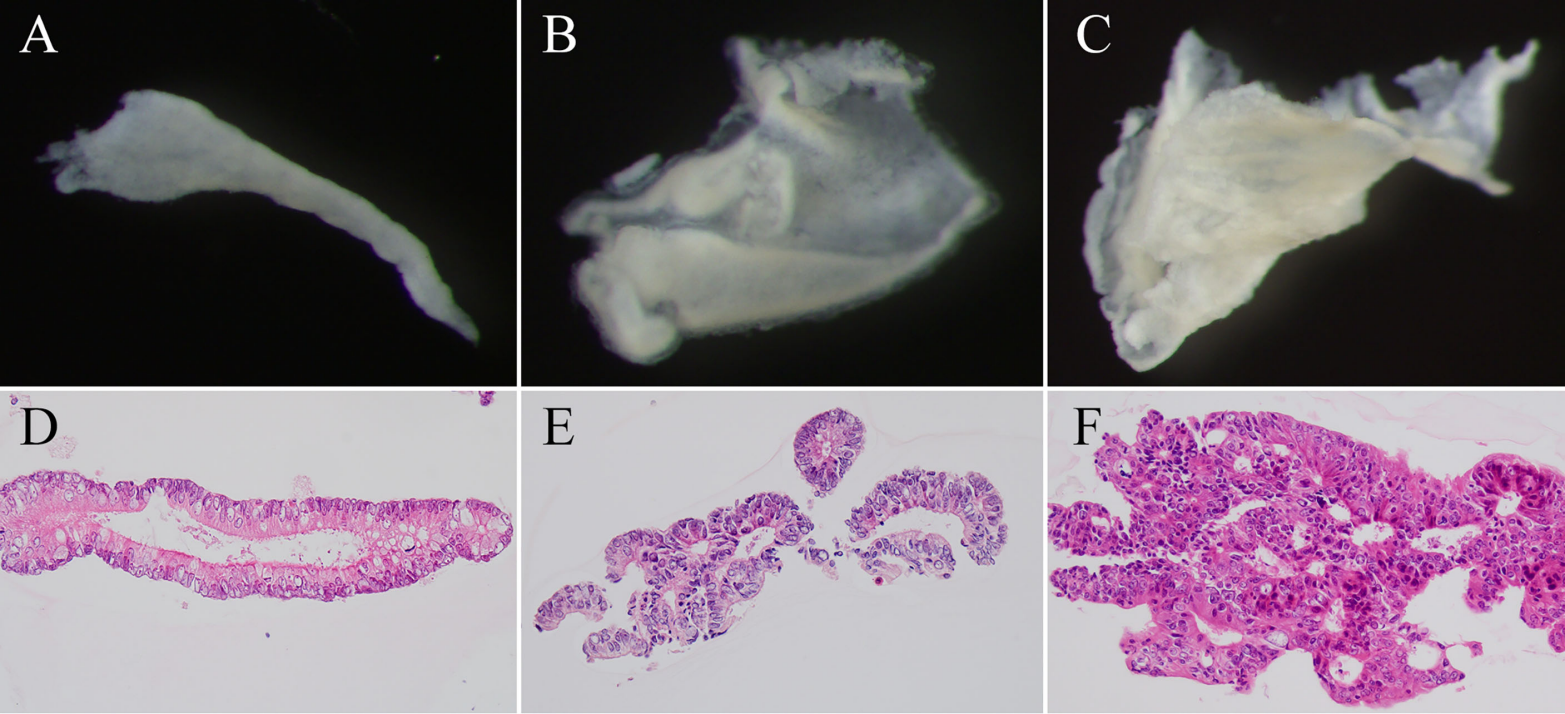
Representative image of isolated crypts. **(A–C)** Isolated glands under a dissecting microscope. **(D–F)** Hematoxylin–eosin staining of isolated crypts. Isolated crypts of low grade tubular adenoma **(A, D)**, intramucosal cancer **(B, E)**, and invasive cancer **(C, F)**.

Crypts were isolated in the current study (18). Briefly, we obtained tumor glands (adenomatous and intramucosal carcinomatous glands) from suspected target lesions based on analysis of magnified observations. By contrast, we collected cancer glands in invasive CRC from the invasive front. Crypt isolation from the tumors and normal mucosa (for surgical specimens, this was the distal site of the colon) was described in previous studies (18, 19). After isolation, crypts were fixed in 70% ethanol and then stored at 4°C until DNA/RNA extraction. After fixation, a dissecting microscope (SZ60; Olympus, Tokyo, Japan) was used to visualize the isolated crypts. A portion of the isolated crypts was subjected to routine processing for histopathological analysis. The samples applied to array-based analyses and validation tests showed no contamination by interstitial cells. Representative images of adenoma, IMC, and invasive cancer are shown in **Figure 1**.

### Nucleic Acid Extraction

#### DNA Extraction

DNA was extracted from tumors and normal glands isolated from each patient by applying a classical phenol-chloroform extraction method. RNeasy Mini kits (Qiagen, Valencia, CA, USA) were to isolate total RNA from tumors and normal glands based on the instructions provided by the manufacturer. Nucleic acid concentrations were measured using a Nanodrop1000 spectrophotometer (Thermo Fisher Scientific, Waltham, MA, USA), and 1.5% denaturing agarose gels were used to determine the purity of the isolated RNA.

#### RNA Extraction

Isolation of total RNA was performed using RNeasy Mini kits (Qiagen, Valencia, CA, USA) according to the manufacturer’s instructions. The nucleic acid concentration was determined using the Nanodrop1000 spectrophotometer (Thermo Fisher Scientific; Waltham, MA, USA), and RNA purity was verified on 1.5% agarose denaturing gels.

### Analysis of Microsatellite Instability

We evaluated the MSI status of the tumor samples using a consensus panel containing five reference microsatellite markers (BAT25, BAT26, D2S123, D3S546, and D17S250) (20). MSI-high was defined as two or more markers being unstable, whereas MSI-low was defined as one marker being unstable; MSS was defined as the absence of instability (21). Normal alleles were typically represented by a major peak accompanied by a few minor peaks. The mobility shift of PCR products from tumor DNA was compared with that obtained from corresponding non-neoplastic crypts. MSI-low was included in MSS status in the present study.

### MiRNA Microarray Analysis

For microarray analysis, we performed polyadenylation and labeling of RNA (200 ng) using a FlashTag Biotin HSR RNA Labeling kit. The RNA was then treated with DNA ligase, hybridized to GeneChip miRNA 4.0 microarrays (Thermo Fisher Scientific) by incubation for 16 h at 48°C, washed, and stained using streptavidin-PE solution. Next, scanning of the stained arrays was carried out using a GeneChip Scanner 3000 7G System (Thermo Fisher Scientific). The Affymetrix miRNA 4.0 microarray used in this study contained 6,631 probes, with 2,570 mature miRNA probes. The methods used here were described in detail in a previous study (22).

To identify candidate miRNAs associated with colorectal tumorigenesis, we then assessed miRNA expression based on the following criteria: less than or greater than 1.5 fold-change in expression compared with normal glands and a *p* value less than 0.05 based on Student’s t-test (without multiple comparison tests).

### Clariom S Human Array and Gene Expression Analysis

Total RNA (500 ng) was applied to a Clariom S Human Array (Thermo Fisher Scientific), which included 21,453 mRNAs. The process, which included probe labeling, chip hybridization, and scanning, was carried out as recommended by the manufacturer. Probe sets (gene-exon) were determined as reported previously (22, 23). Transcriptome Analysis Console (TAC, version 4.0.1.36) was used to generate array data, and Affymetrix Chromosome Analysis Suite v.4.1 (Affymetrix Inc., USA) was used for analysis. mRNA expression was evaluated using low statistical stringency (without multiple comparison tests), as described above for the evaluation of miRNA expression (23).

### Ingenuity Pathways Analysis

IPA (Ingenuity System Inc., USA, http://www.ingenuity.com) is an approach for identifying interactions between miRNAs and their mRNA targets. The “microRNA target filter” function in IPA was used to build graphical models of the molecular relationships between mRNAs and miRNAs showing significant changes in expression in isolated tumor glands of each colorectal tumor compared with isolated normal glands. miRNA/mRNA interactions with high confidence (predicted), moderate confidence (predicted), or confirmed experimental observations were selected.

### Simple Linear Regression Analysis

Simple linear regression analysis was used to further analyze candidate mRNAs and their targeting miRNAs along with corresponding expression profiles from microarray analyses. The significance of regression coefficients was tested based on 95% confidence intervals (CIs). If the 95% CI for a coefficient did not include zero, there was less than a 5% chance that the coefficient was zero (p < 0.05). Thus, such coefficients were considered significant in the model.

### RT- Quantitative PCR for Validation Analyses

Microarray results were validated using RT-qPCR. First-strand cDNA was generated by reverse transcription of 1 μg total RNA using the Qiagen cDNA Synthesis Kit (Qiagen). The resulting cDNA was then applied as a template for qPCR using gene-specific primers, using β-actin as the housekeeping control gene (control of each mRNA). RT-qPCR was performed using Power SYBR Green PCR Master Mix (Thermo Fisher Scientific) on the CFX96 Touch Real-Time PCR Detection System (Bio-Rad, Hercules, CA, USA). The 2^−Δ^ *^Ct^* method was used to calculate relative gene expression levels based on the Livak and Schmittgen method (24). The expression levels of each miRNA were compared with that of RNU6B. β-actin and RNU6B are used as a control of mRNA and miRNA standardly. The primer sequences are listed in **Supplementary Table 1**.

### Transfection With miRNA Mimics

CRC cells (CaCO2, Colo320, DLD1, HCT116, HT29, RKO, SW48, SW480, and SW620 cell lines) at 5 × 10^5^/well in 6-well plates were transfected with 40 pmol of the following mirVana miRNA mimics (Thermo Fisher Scientific): *miRNA-6078-5p* (assay ID: MC28605), *miRNA 3064-5p* (assay ID: MC20900), and Negative Control #1. Transfection was performed using Lipofectamine RNAiMAX (Thermo Fisher Scientific) according to the manufacturer’s instructions. After incubation for 72 h, total RNA was extracted using the RNeasy Mini kit (Qiagen) according to the manufacturer’s instructions.

### Statistical Analysis

Differences in miRNA and mRNA expression levels were evaluated using limma (*p* values without adjusted Benjamini–Hochberg FDR correction to identify candidate miRNAs and mRNAs from array-based analyses). The correlation of miRNA with mRNA was examined using a single regression analysis. We used statistical software for analysis (JMP pro 13.0 software package for Windows (SAS Institute Inc., Cary, NC, USA). Results with a *p* value less than 0.05 were considered significant.

### Work Flow (**Supplementary Figure 1**)

First, we examined the differences in mature miRNA expression levels between the colorectal tumors examined (adenoma, IMC, and MSS CRC) and normal glands using the following criteria: fold-change expression > |1.5| and p < 0.05 for the GeneChip miRNA 4.0 microarray and Clariom S human array data. Second, we assessed the interactions between miRNAs and mRNAs using the microRNA Target Filter in IPA to identify potential candidate mRNA/miRNA pairs based on the arrays. Third, simple regression analysis was performed. Fourth, validation analyses including RT-qPCR were performed in cohort 2. In addition, transfection assays were also conducted. Finally, the expression of candidate mRNAs and miRNAs in TCGA database was evaluated.

To examine the associations between dysregulated miRNAs and mRNAs, we evaluated the inverse correlations between miRNAs and mRNAs in adenomatous and carcinomatous lesions within the same tumor using the same method as above. However, validation analyses were not performed.

## Results

### Microsatellite Analyses of Colorectal Adenomas, IMCs, and Invasive CRC

In cohort 1, only three tumors were classified as MSI-low and the remaining as MSS. Thus, all of the samples were categorized as MSS based on previously reported criteria (20). Representative Figure is depicted in **Supplementary Figure 2**.

### Analyses of Comprehensive Expression Patterns of miRNAs and mRNAs in Colorectal Tumors (Adenomas, IMCs, and CRC) Having an MSS Phenotype

We examined 15 adenomas, 8 IMCs, and 19 CRCs using array-based analysis.

#### miRNA Expression Profiling in Colorectal Tumors

**Figure 2:**
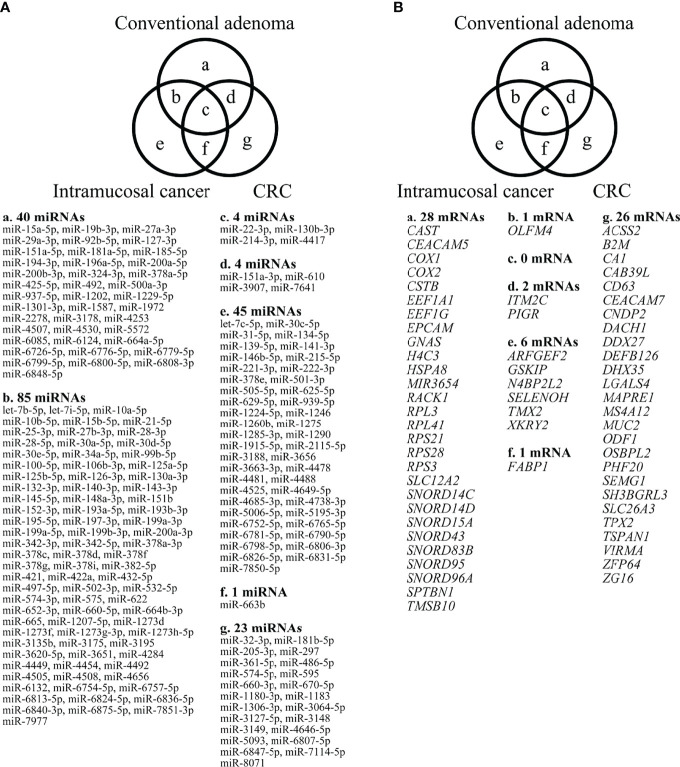
Differential expression of miRNAs, comparing adenomas, IMCs, and CRCs having the MSS phenotype, as depicted by Venn diagram analysis. The numbers of miRNAs identified in adenomas, IMCs, and CRCs having the MSS phenotype were 40, 45 and 23, respectively. Among the three lesions, a considerable number of miRNAs overlapped with each other (adenoma and IMC, 85 miRNAs; adenoma and CRC with an MSS phenotype, 4 miRNAs; IMCs and CRCs having the MSS phenotype, 1 miRNA; adenomas, IMCs, and CRCs having the MSS phenotype, 4 miRNAs). 1-B. Differential expression of mRNAs between adenomas, IMCs, and CRCs having the MSS phenotype, as depicted by Venn diagram analysis. The numbers of mRNAs identified in adenomas, IMCs, and CRCs having the MSS phenotype were 28, 6, and 26. Among the three lesions, few mRNAs overlapped (adenoma and IMC, 1 mRNA; adenoma and CRC with an MSS phenotype, 2 mRNAs; IMCs and CRCs having the MSS phenotype, 1 mRNA; adenomas, IMCs, and CRCs having the MSS phenotype, none).

In our analysis of expression levels in isolated adenomatous and normal crypts, we identified 133 miRNAs showing differential expression (31 upregulated and 102 downregulated miRNAs). Then, we evaluated the expression levels of miRNAs in isolated IMC glands and compared the results with those from normal isolated crypts; the results identified a total of 135 differentially expressed miRNAs, including 21 upregulated miRNAs and 114 downregulated miRNAs. Finally, we compared the expression levels of miRNAs in isolated cancer (CRC with an MSS phenotype) and corresponding normal glands and found 32 differential expressions (29 upregulated and 3 downregulated). The detailed data are displayed in a Venn diagram in **Figure 2-A**.

#### mRNA Expression Profiling of Colorectal Tumors

First, we assessed differences in mRNA expression levels between isolated neoplastic and normal glands. Consequently, we found 31 differentially upregulated mRNAs in isolated adenomatous samples. Similarly, we identified 21 upregulated mRNAs and 114 downregulated mRNAs from isolated IMC glands. Finally, we found changed expression levels in 32 mRNAs in isolated invasive cancer glands (29 upregulated and 3 downregulated). The detailed data based on a Venn diagram were previously described (**Figure 2-B**) (23).

#### Integrative Analysis of miRNA and mRNA Expression Levels and Simple Regression Analysis for Array-Based Assay Samples

We then evaluated the associations between opposite expression levels of miRNA/mRNA using IPA. Fifty-seven miRNA/mRNA pairs had inverse expression patterns in the adenoma samples (**Supplementary Figure 3-A**). Moreover, 12 and 23 miRNA/mRNA pairs showed inverse expression patterns in IMC (**Supplementary Figure 3-B**) and CRC with the MSS phenotype (**Supplementary Figure 3-C**), respectively.

Simple regression analysis of the array data was performed to select candidate miRNA/mRNA pairs. According to these criteria, the following five pairs of miRNAs/mRNAs were retained among 57 pairs in the adenoma samples (**Supplementary Figure 4-A**; **Supplementary Table 2-A**): *hsa-miRNA-34a-5p/SLC12A2* (solute carrier family 12 member 2), *hsa-miRNA-15b-5p/SLC12A2*, *hsa-miRNA-195-5p/SLC12A2*, *hsa-miRNA-15a-5p/SLC12A2*, and *hsa*-*miRNA-3907*/*EEF1A1*. Moreover, in the IMC samples (**Supplementary Figure 4-B** **;** **Supplementary Table 2-B**), among 12 pairs of miRNA/mRNA networks, we identified 2 pairs of miRNA/mRNA, including *hsa-miRNA-664b-3/GSKIP* (glycogen synthase kinase 3 Interacting Protein) and *hsa-miRNA-502-3p/*olfactomedin 4 (*OLFM4*). On the other hand, among 23 pairs of miRNA/mRNA in CRC with an MSS phenotype, 3 pairs were selected. They included *hsa-miRNA-5093/SLC26A3*, *hsa-miRNA-6807-5p/ZG16* (Zymogen granule protein 16) and *hsa-miRNA- 3064-5p/SH3BGRL3* (Src homolog 3 Domain Binding Glutamate Rich Protein Like 3) (**Supplementary Figure 4-C** **;** **Supplementary Table 2-C**).

#### Simple Regression Analysis of the Validation Cohort

**Figure 3:**
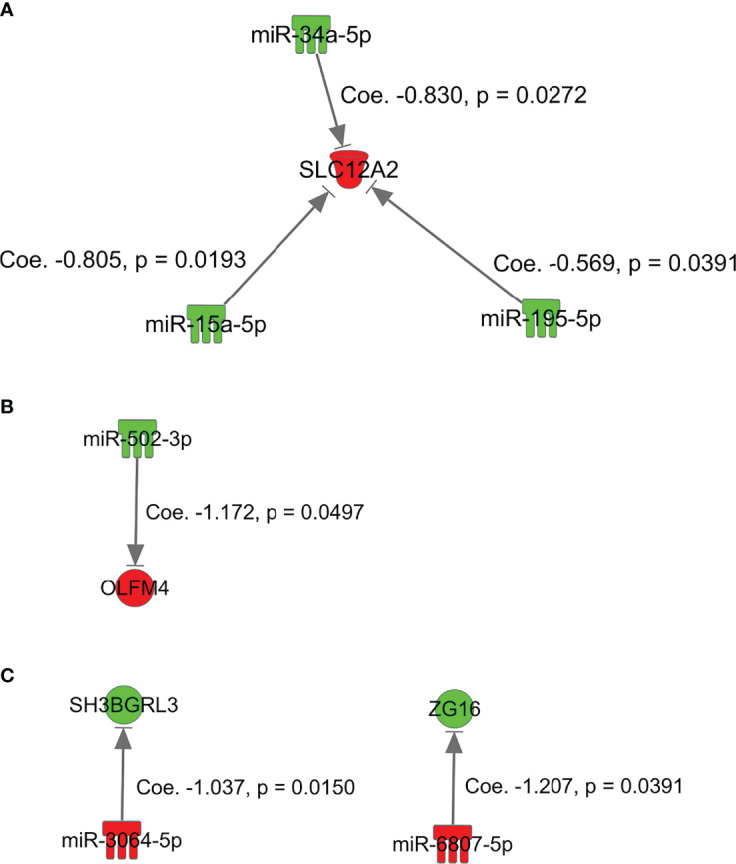
miRNAs and mRNAs with inverse expression patterns in array-based assays in the first cohort of adenoma, intramucosal cancer, and CRC with MSS. **(A)**, *SKC12A2/miRNA-15a-5p, miRNA-34a-5p*, and *miRNA-195-5p* in adenoma. **(B)** GSKIP/miRNA-664b-3p and OLFM4/miRNA-502-3p in IMC. **(C)** *hsa-miRNA-6807-5p/ZG16* and *hsa-miRNA 3064-5p/SH3BGRL3* in MSS CRC.

We validated the results that were obtained from the first cohort (array-based assays) using a real-time PCR method in 37 colorectal tumors (15 adenomas, 8 IMCs, and 14 CRCs with an MSS phenotype). In isolated adenoma samples, among 5 paired miRNAs/mRNAs, 3 pairs (*hsa-miR-34a-5p/SLC12A2*, *hsa-miR-15a-5p/SLC12A2*, and *hsa-miR-195-5p/SLC12A2*) were retained in single regression analysis (**Figure 3-A**; **Table 2-a**). Among the 2 miRNA/mRNA pairs in the IMC samples, the expression level of *hsa-miRNA-502-3p* was inversely correlated to that of *OLFM4* (**Figure 3-B** **;** **Table 2-b**). In isolated invasive carcinomatous gland samples, 2 pairs were retained (**Figure 3-C** **;** **Table 2-c**): *hsa-miRNA-6807-5p/ZG16* (zymogen granule protein 16) and *hsa-miRNA 3064-5p/SH3BGRL3*.

**Table 2-A:** Simple regression analysis between micro RNA and mRNA in conventional adenoma in validation cohort (cohort 2).

mRNA	micro RNA	Regression coefficient	Adjusted coefficient of determination	95% CI	*p*-value
*SLC12A2*	miR-15a-5p	-0.805	0.304	[-1.456, -0.153]	0.0193
*SLC12A2*	miR-34a-5p	-0.830	0.270	[-1.551, -0.109]	0.0272
*SLC12A2*	miR-195-5p	-0.569	0.233	[-1.105, -0.033]	0.0391
*SLC12A2*	miR-15b-5p	-0.673	0.040	[-1.828, 0.482]	0.2304
*EEF1A1*	miR-3907	1.095			

CI, Confidence Interval.

**Table 2-B:** Simple regression analysis between micro RNA and mRNA in intramucosal cancer in validation cohort (cohort 2).

mRNA	micro RNA	Regression coefficient	Adjusted coefficient of determination	95% CI	*p*-value
*OLFM4*	miR-502-3p	-1.172	0.417	[-2.342, -0.002]	0.0497
*GSKIP*	miR-664b-3p	0.738			

CI, Confidence Interval.

**Table 2-C:** Simple regression analysis between micro RNA and mRNA in colorectal cancer with an MSS phenotype in validation cohort (cohort 2).

mRNA	micro RNA	Regression coefficient	Adjusted coefficient of determination	95% CI	*p*-value
*SH3BGRL3*	miR-3064-5p	-1.037	0.351	[-1.834, -0.24]	0.0150
*ZG16*	miR-6807-5p	-1.207	0.251	[-2.343, -0.071]	0.0391
*SLC26A3*	miR-5093^*^				

MSS, microsatellite stable; CI, Confidence Interval; *, no amplification.

### Comprehensive Analysis of Expression Pattern of Micro RNA and Messenger RNA Within the Same Tumor

We aimed to identify genome-wide miRNA/mRNA expression patterns occurring within the same tumor during neoplastic progression according to the adenoma–carcinoma sequence.

### miRNA Expression Profiling Within the Same Tumor

**Figure 4:**
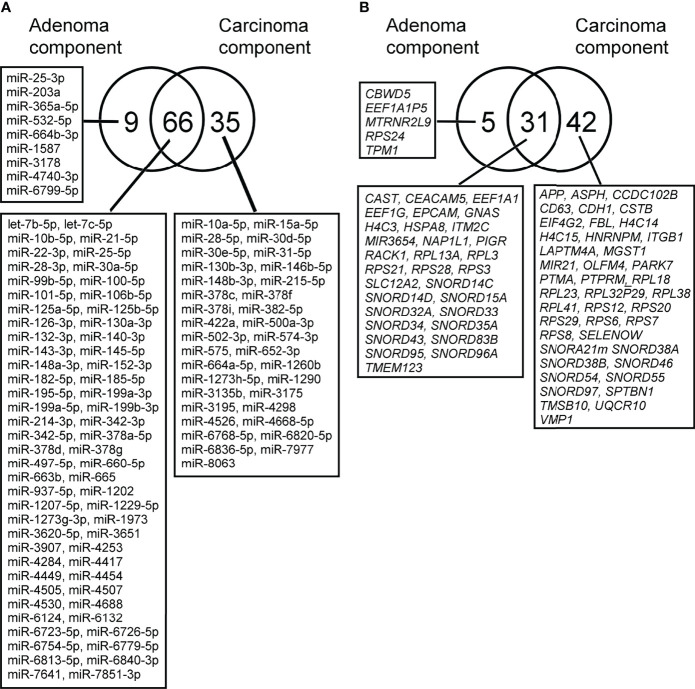
**(A)**. Differential expression of miRNAs between isolated adenoma and carcinomatous glands samples obtained from “cancer in/with adenoma” case, as depicted by Venn diagram analysis. The numbers of miRNAs identified in adenoma, and cancer glands were 9 and 35, respectively. Among the two lesions, a considerable number of miRNAs overlapped with each other (adenoma and cancer, 31 miRNAs). **2-B**. Differential expression of mRNAs between isolated adenoma and carcinomatous glands samples obtained from “cancer in/with adenoma” case, as depicted by Venn diagram analysis. The numbers of mRNAs identified in adenoma and cancer samples were 5, 31, and 42. Among the two lesions, a considerable number of mRNAs were overlapped (adenoma and cancer samples, 31 mRNAs).

Using a similar method, we also investigated the expression profiles of miRNAs occurring in isolated carcinomatous glands within the same tumor. First, in a comparison of expression levels between isolated adenomatous/carcinomatous tumors and normal crypts, we found that 75 miRNAs were differentially expressed (32 upregulated and 43 downregulated) in isolated adenomatous glands and 101 differentially expressed miRNA (37 upregulated and 64 downregulated) in isolated carcinomatous glands (**Figure 4-A**).

### mRNA Expression Profiling in Isolated Adenomatous and Carcinomatous Glands Within the Same Tumor

Global mRNA expression profiles of 15 isolated colorectal adenomatous and carcinomatous glands within a tumor were evaluated and compared with those of normal isolated gland samples. In isolated adenomatous samples, 36 mRNAs showed differential upregulation, and 71 upregulated and 2 downregulated mRNAs were then set from isolated carcinomatous glands within a tumor. The detailed data and a Venn diagram were previously reported (**Figure 4-B**) (24).

### Integrated Analysis of miRNA and mRNA Expression Within the Same Tumor and Simple Regression Analysis of Array-Based Samples

Next, IPA was used to identify functional miRNA-mRNA patterns by evaluating the inverse expression of miRNAs and their targeted mRNAs. Of 75 aberrantly expressed miRNAs identified in isolated adenomatous glands, 39 miRNA-mRNA pairs showed opposite expression patterns (**Supplementary Figure 5-A** **;** **Supplementary Table 3-A**). Of 101 aberrantly expressed miRNAs set from isolated carcinomatous glands, 123 miRNA-mRNA pairs with opposite expression patterns were suggested (**Supplementary Figure 5-B** **;** **Supplementary Table 3-B**).

**Figure 5:**
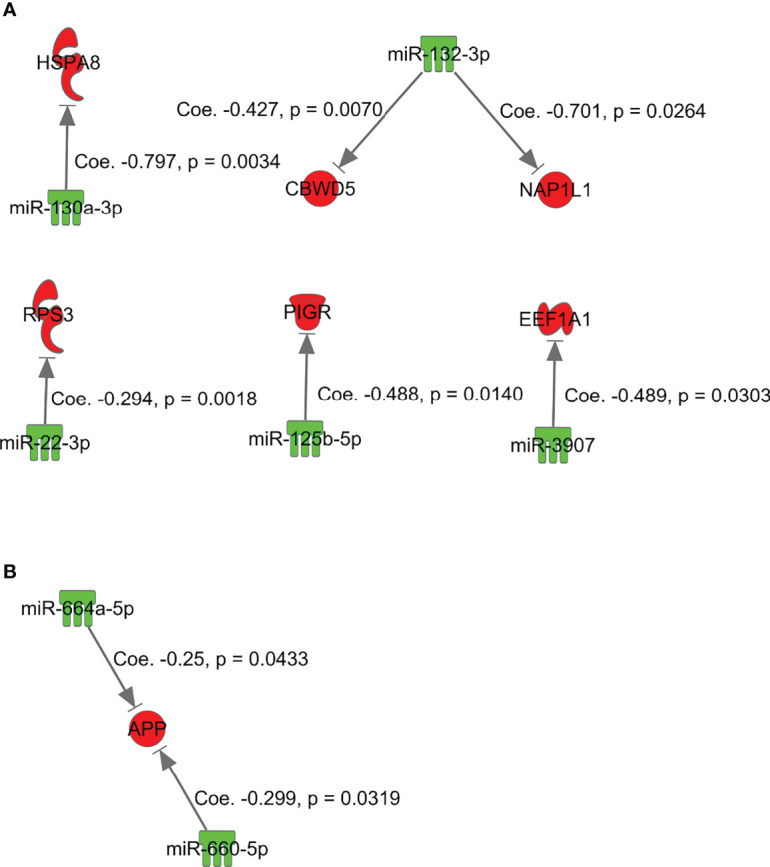
miRNAs and mRNAs with inverse expression patterns in the adenoma component in the array-based assays. **(A)** *miRNA-132-3p/CBWD5*, *miRNA-3907/EEF1A1*, *miRNA-130a-3p/HSPA8*, *miRNA-132-3p/NAP1L1*, *miRNA-125b-5p/PIGR*, and *miRNA-22-3p/RP53*. **(B)** *miRNA-660-5p* and *miRNA-664a-5p/APP*.

Simple regression analysis for array-based assays was performed to select candidate pairs of miRNA-mRNA networks (**Figures 5-A and -B**). According to these criteria, we found 6 miRNA/mRNA pairs, including *miRNA-132-3p/CBWD5* (cobalamin synthase W domain-5), *miRNA-3907/EEF1A1*, *miRNA-130a-3p/HSPA8* (heat shock protein family A [Hsp70] member 8), *miRNA-132-3p/NAP1L1* (nucleosome assembly protein 1 like 1), *miRNA-125b-5p/PIGR* (polymeric immunoglobulin receptor), and *miRNA-22-3p/RP53* (retinitis pigmentosa 53) in isolated adenomatous glands (**Figure 5-A**). We also identified 2 miRNA/mRNA pairs, including *miRNA-660-5p* and *miRNA-664a-5p/APP* (amyloid beta precursor protein), in isolated carcinomatous glands (**Figure 5-B**).

### Effects of the Transfected miRNA Mimics *miRNA-6078-5p* and *miRNA 3064-5p* on *SH3BGRL3* and *ZG16* Expression

**Figure 6:**
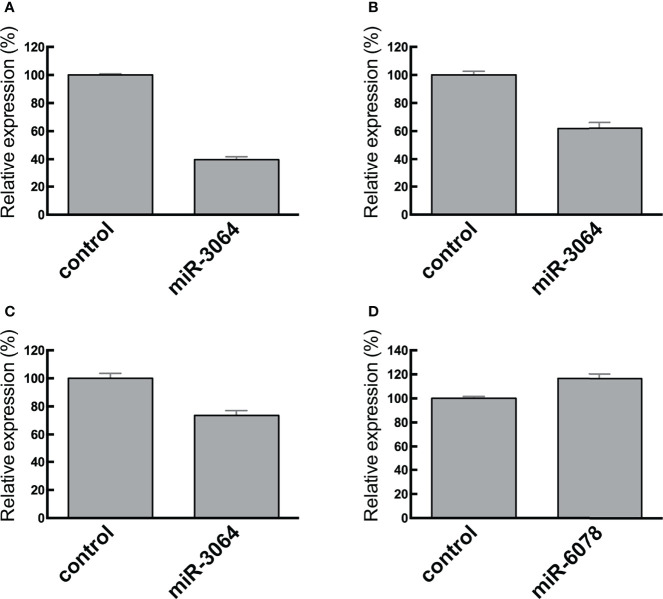
RT-qPCR analysis of *SH3BGRL3* **(A–C)** and *ZG16* **(D)** in normal colon tissues and CRC cell lines. Relative reduced expression of *SH3BGRL3* in DLD1 cells after transfection of the miR-3064 mimic. **(B)** Relative reduced expression of *SH3BGRL3* in RKO cells after transfection of the miR-3064 mimic. **(C)** Relative reduced expression of *SH3BGRL3* in Colo320 cells after transfection of the miR-3064 mimic. **(D)** Relative high expression of *ZG16* in CaCO2 cells after transfection of the miR-6078 mimic.

We transfected CRC cells with the miRNA mimics *miRNA-6078-5p* and *miRNA 3064-5p* and assessed their effects on the expression of candidate target genes (**Figure 6**). We found that ectopic expression of *miRNA 3064-5p* suppressed *SH3BGRL3* expression. On the other hand, ectopic expression of *miRNA-6078-5p* had no effect on *ZG16* expression; however, this result does not necessarily imply that *ZG16* is not a potential target gene of *miRNA-6807-5p* in CRC cells.

### Associations Between ZG16 and hsa-miRNA 3064-5p and Between SH3BGRL3 and hsa-miRNA-6807 Expression in The Cancer Genome Atlas Database

We attempted to explore miRNA and mRNA expression data from the portal site of the TCGA-colon adenocarcinoma project database (RNA-Seq data, HTSeq-Counts and miRNA-Seq data, and British Canadian’s Genome Science Center miRNA profiling data). However, in this database, we could not find public data examining the association between *ZG16* and *hsa-miRNA-6807* or between *SH3BGRL3* and *hsa-miRNA 3064-5p* expression.

## Discussion

Bioinformatics and data mining are rapidly advancing, thereby allowing a multi-omics approach to the investigation of the biological alterations underlying disease (25). New technologies now enable the use of large-scale data bases. The Cancer Genome Atlas is a huge database of cancer-related results and has been used as a reference for studies performed worldwide (6). By contrast, MiRTarBase provides data for experimentally confirmed miRNA-mRNA pairs in several diseases (26). However, relatively few studies have targeted pairs of miRNAs/mRNAs identified in the “adenoma-carcinoma sequence”. Therefore, we examined potential network patterns of miRNAs/mRNAs occurring in adenomas, IMCs, and CRCs having the MSI phenotype based on a comprehensive genome-wide method.

In the present study, two approaches were adopted to evaluate neoplastic progression from adenomatous to carcinomatous lesions. First, comprehensive expression analyses of miRNAs and mRNAs were separately performed in each lesion, including adenomas, IMCs, and CRCs having the MSS phenotype (model A). Second, using a similar method, expressions of miRNA and mRNA were examined separately in isolated adenomatous and carcinomatous glands obtained from the same tumor (model B). Similar pairs of miRNAs/mRNAs are expected to be found in both models. However, the patterns of miRNAs/mRNAs found differed between models A and B. In model B, this finding is very important in that molecular alterations occurring in the adenoma component without carcinomatous lesions (adenoma only lesion) differ from those derived from the adenoma component with carcinomatous lesions within a single tumor. This theory may be supported by the finding that multiple networks of miRNAs/mRNAs are closely associated with model B lesions, unlike the network pattern in model A (27, 28).

In the present study, some mRNA/miRNA pairs with an inverse correlation were found to be closely associated with the initial stage of colorectal carcinogenesis. The translocation of substrates through biological membranes is regulated by transporter proteins (29), including ATP-binding cassette and solute carrier (SLC) proteins, which are expressed in most tissues (29). Importantly, SLC proteins have been shown to exhibit differential expression in gastrointestinal neoplasms (29). In gastrointestinal tumors, including colorectal adenomas, the functions of *SLC12A2* have not been fully elucidated. Here, the expression of *SLC12A2* was upregulated due to downregulation of several miRNAs, including *hsa-miR-34a-5p*, *hsa-miR-15b-5p*, and *hsa-miR-195-5p*, in isolated adenomatous glands derived from model A lesions. These results suggest that *SLC12A2* could contribute to the initial stages of colorectal tumorigenesis.

In the present study, expression of *hsa-miRNA-502-3p* was inversely correlated to that of *OLFM4* in IMC. OLFM4 is an evolutionarily conserved glycoprotein that belongs to the OLFM family and is abundantly expressed in intestinal crypts (30). A recent study also demonstrated that OLFM4 plays an important role in preserving intestinal Lgr5-positive stem cells (31). This result suggests that reduced expression of *OLFM4*, which is regulated by high expression of *miRNA-502-3p*, may be closely associated with early colorectal carcinogenesis.

In the advanced stage of colorectal carcinogenesis, there was an inverse correlation between *miRNA-6807-5p* and *ZG16* and between *miRNA 3064-5p* and *SH3BGRL3*. *ZG16* is one of the most significantly downregulated genes in colorectal cancer (CRC) tissues (32, 33). Previous studies showed that reduced expression of *ZG16* may play an important role in cancer progression (32, 33). In addition, *ZG16* may serve as a potential biomarker for CRC diagnosis and prognosis (33). According to the transfection assay in our study, however, we did not find that downregulation of *ZG16* was correlated with upregulation of *miRNA-6807-5p.* There are a couple of explanations for this result. First, *ZG16* may not be a direct target of *miRNA-6807-5p*, and another mechanism may play an important role in *ZG16* regulation. Second, the results of our *in vitro* experiment may not reflect what occurs *in vivo*. On the other hand, we identified that ectopic expression of *miRNA 3064-5p* suppressed *SH3BGRL3* expression. We suggest that the inverse relationship between *miRNA 3064-5p* and *SH3BGRL3* plays an important role in colorectal carcinogenesis.

Our findings suggested inverse correlations between paired mRNAs and miRNAs necessary for progression of a tumor from adenoma to IMC (model B). In adenomatous lesions isolated from the same tumor, we observed two inverse correlations of paired mRNAs/miRNAs, including *RP53/miRNA-3907* or *miRNA22-3p* and HSPA8/*miRNA-130a-3p*. First, ribosomal protein (RP) expression has been shown to be higher in tumor cells than in normal cells (21, 34). RPs are differentially expressed in tumors, and the expression levels vary among the different stages of cancer. RPs may promote tumor development in CRC (21, 34). Second, HSPA8 protein is a constitutively expressed molecular chaperone that assists the conformational folding or unfolding of other macromolecular structures (35). A previous study showed that upregulation of HSPA8 plays a vital role in the development of endometrial carcinoma (36). According to the present results, however, such transcripts may act as tumor suppressors in adenoma development. We suggest that the mRNA/miRNA pairs HSPA8/*miRNA-130a-3p* and *RP53/miRNA-3907* or *miRNA22-3p* play an important role in the development of the adenomatous component of a tumor.

We also identified two miRNA/mRNA pairs, including *miRNA-660-5p* and *miRNA-664a-5p/APP*, in isolated carcinomatous glands from the same tumor (model B). *APLP2* shows a broad expression pattern, whereas *APLP1* is expressed primarily in neural tissues (37, 38). APP protein and *APLP2* mRNA are overexpressed in many tumors (37), including cancers of the prostate, breast, colon, thyroid, lung, nasopharynx, and gastrointestinal tract (37). Both *in vitro* and *in vivo* studies have demonstrated the roles of APP in promoting colon cancer growth and proliferation (39). In the present study, however, APP expression was downregulated by high expression of *miRNA-660-5p* and *miRNA-664a-5p*, suggesting that the associations of *miRNA-660-5p* and *miRNA-664a-5p* with *APP* may promote tumor progression from adenoma to carcinoma.

This study had some limitations. First, we evaluated only a small number of samples in this study. However, we used isolated tumor glands, thereby excluding interstitial cells that could otherwise obscure molecular alterations within tumor cells. Isolated tumor glands are essential in the search for molecular alterations in the target tissue. Thus, our data may provide new insights into the evaluation of colorectal carcinogenesis, especially at the early stage, which has not been fully identified previously. Second, we could not perform a validation test in model B lesions. Importantly, obtaining isolated adenomatous and carcinomatous glands from the same tumor is quite challenging when analyzing clinical samples. As far as we know, the analysis of the expression of miRNA and mRNA was first reported in model B. We provided additional information elucidating neoplastic progression within the same tumor.

In conclusion, we examined the relationship between miRNA and mRNA expression in isolated adenomatous and carcinomatous glands, including IMC and CRC with an MSS phenotype. In addition, we identified miRNA/mRNA networks in the progression from adenomatous to carcinomatous lesions by analyzing the association between miRNA and mRNA expression in the same tumor. It is particularly interesting that network patterns of miRNA/mRNA in adenoma and IMC were different from those of adenomatous and carcinomatous lesions within a single tumor. These results indicated that there are two types of adenomas (molecularly stable and unstable). Further studies are needed to identify network patterns of miRNA/mRNA expression and function in colorectal tumors.

## Data Availability Statement

The datasets presented in this study can be found in online repositories. The names of the repository/repositories and accession number(s) can be found in the article/**Supplementary Material**.

## Ethics Statement

The studies involving human participants were reviewed and approved by The ethics committee of Iwate Medical University Hospital (reference number: HG2020-067). The patients/participants provided their written informed consent to participate in this study. Written informed consent was obtained from the individual(s) for the publication of any potentially identifiable images or data included in this article.

## Author Contributions

TS (first and corresponding author) contributed to manuscript preparation, including data collection and analysis. MO and RS carried out data collection and statistical analyses. ME and YT collected isolated tumors and normal glands. NY assisted with data preparation. KO, AS, and TM gave clinical support during manuscript preparation. TN and HS supported the molecular analyses. All authors contributed to the article and approved the submitted version.

## Conflict of Interest

The authors declare that the research was conducted in the absence of any commercial or financial relationships that could be construed as a potential conflict of interest.

## Publisher’s Note

All claims expressed in this article are solely those of the authors and do not necessarily represent those of their affiliated organizations, or those of the publisher, the editors and the reviewers. Any product that may be evaluated in this article, or claim that may be made by its manufacturer, is not guaranteed or endorsed by the publisher.

## Supplementary Material

Supplementary Figure 1 Work flow of the present study.

Supplementary Figure 2
**(A)**. Histological feature (HE). **(B)**. Isolated glands under a dissecting microscope. **(C)**. Hematoxylin–eosin staining of isolated crypts. **(D)**. Normal gland (BAT25). **(E)**. Tumor gland (BAT25). **(F)**. Normal gland (BAT26). **(G)**. Tumor gland (BAT26).

Supplementary Figure 3 Candidate connection of paired miRNAs/mRNAs with reciprocal expression in adenoma **(A)**, IMC **(B)**, and CRC with the MSS phenotype **(C)** (type A).

Supplementary Figure 4 Significant miRNA/mRNA pairs with inverse relationships in adenoma **(A)**components **(A)**, IMC **(B)**, and CRC with the MSS phenotype **(C)**.

Supplementary Figure 5 miRNA/mRNA pairs with inverse relationships based on the adenoma–carcinoma sequence within the same tumor. **(A)** Adenoma and **(B)** IMC components.
