# Supplementary figures and images for: Genome-Wide Analysis of microRNA and mRNA Expression in Colorectal Intramucosal Neoplasia and Colorectal Cancer With a Microsatellite-Stable Phenotype Based on Adenoma-Carcinoma Sequences
Source: Front Oncol. 2022 Jul 7;12:831100. doi: 10.3389/fonc.2022.831100 (PMC9300861; doi:10.3389/fonc.2022.831100)

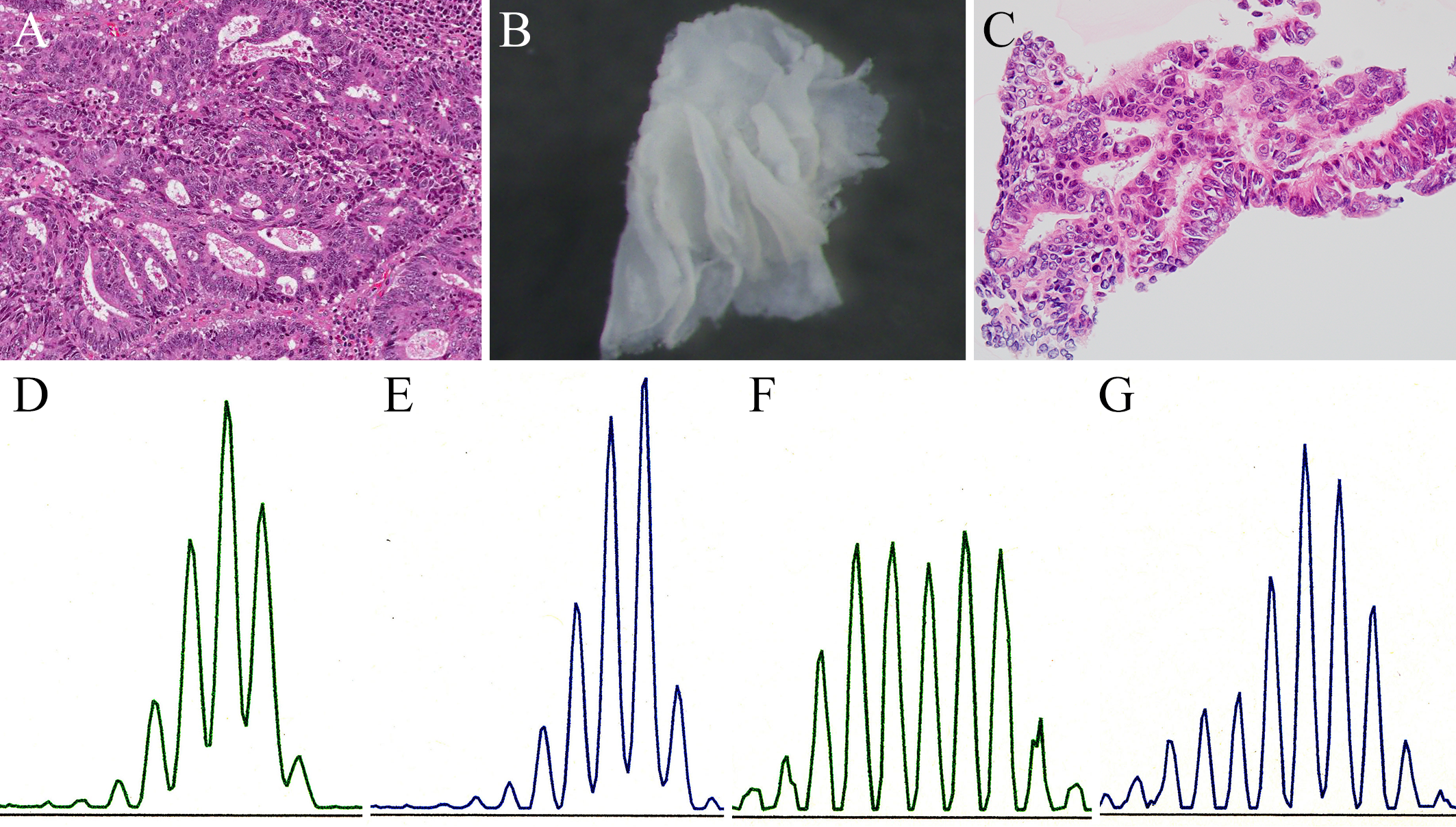

Supplement: Supplementary Figure 1 — Work flow of the present study. [file Image_1.tif]

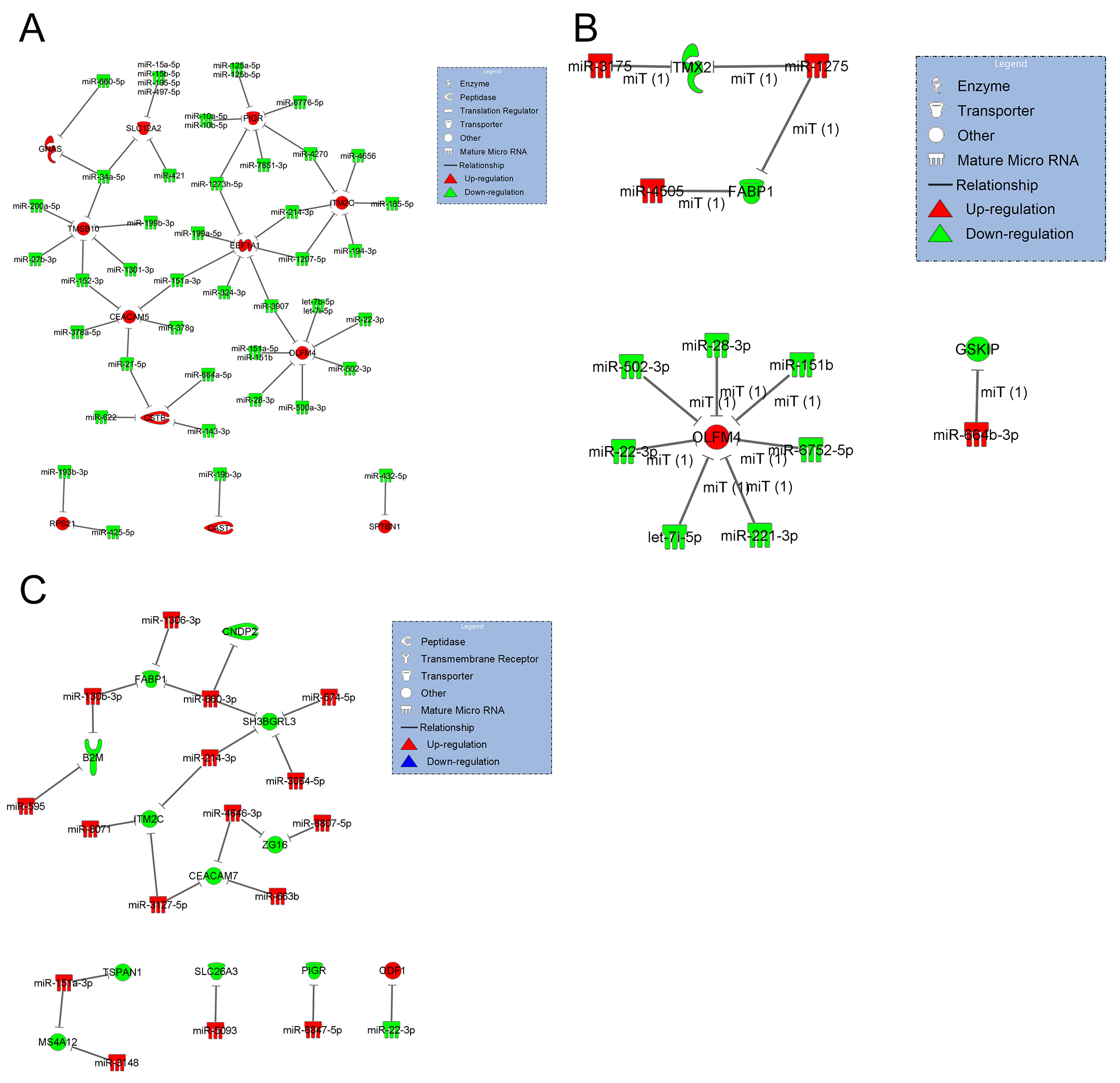

Supplement: Supplementary Figure 2 — (A). Histological feature (HE). (B). Isolated glands under a dissecting microscope. (C). Hematoxylin–eosin staining of isolated crypts. (D). Normal gland (BAT25). (E). Tumor gland (BAT25). (F). Normal gland (BAT26). (G). Tumor gland (BAT26). [file Image_2.tif]

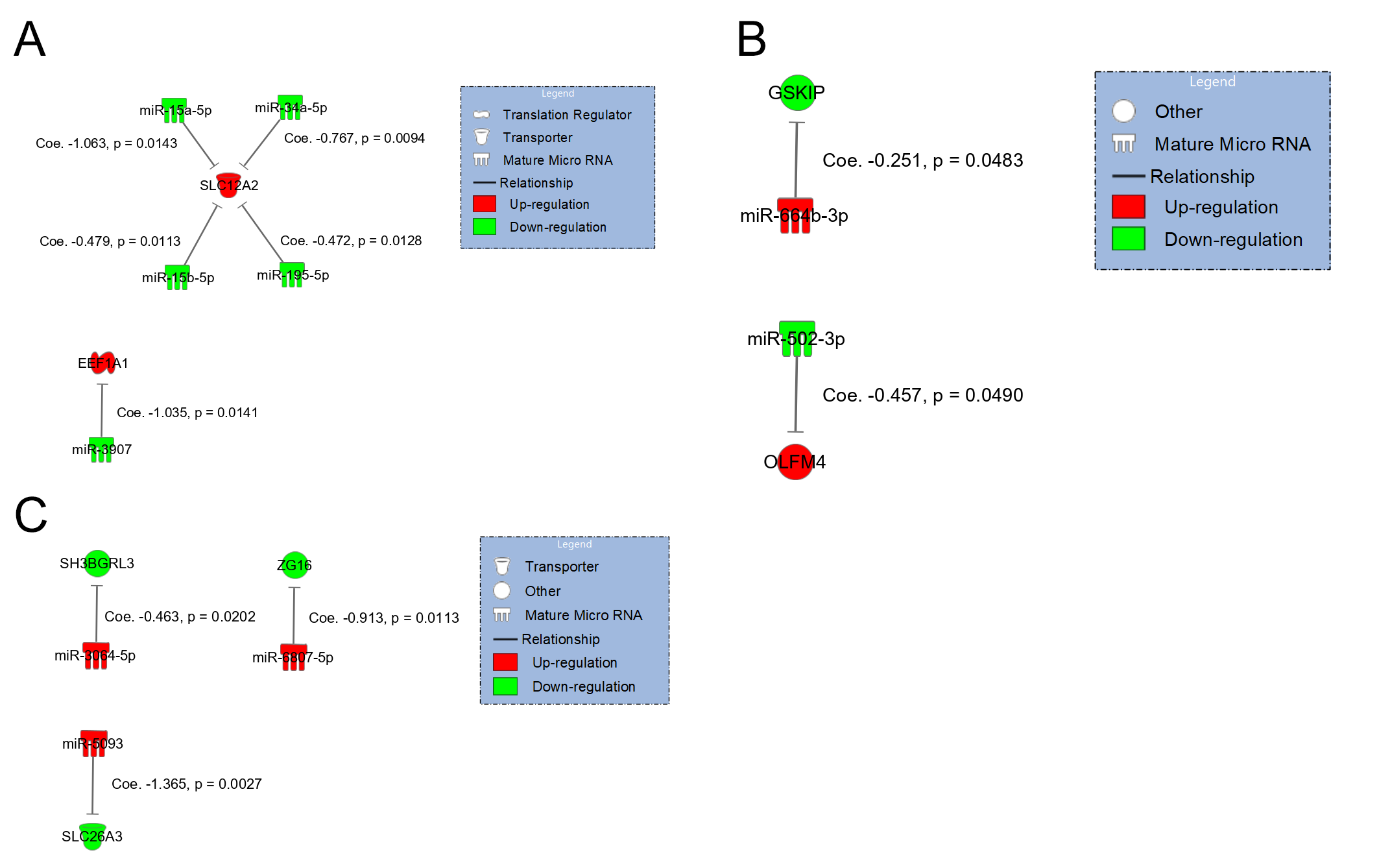

Supplement: Supplementary Figure 3 — Candidate connection of paired miRNAs/mRNAs with reciprocal expression in adenoma (A), IMC (B), and CRC with the MSS phenotype (C) (type A). [file Image_3.tif]

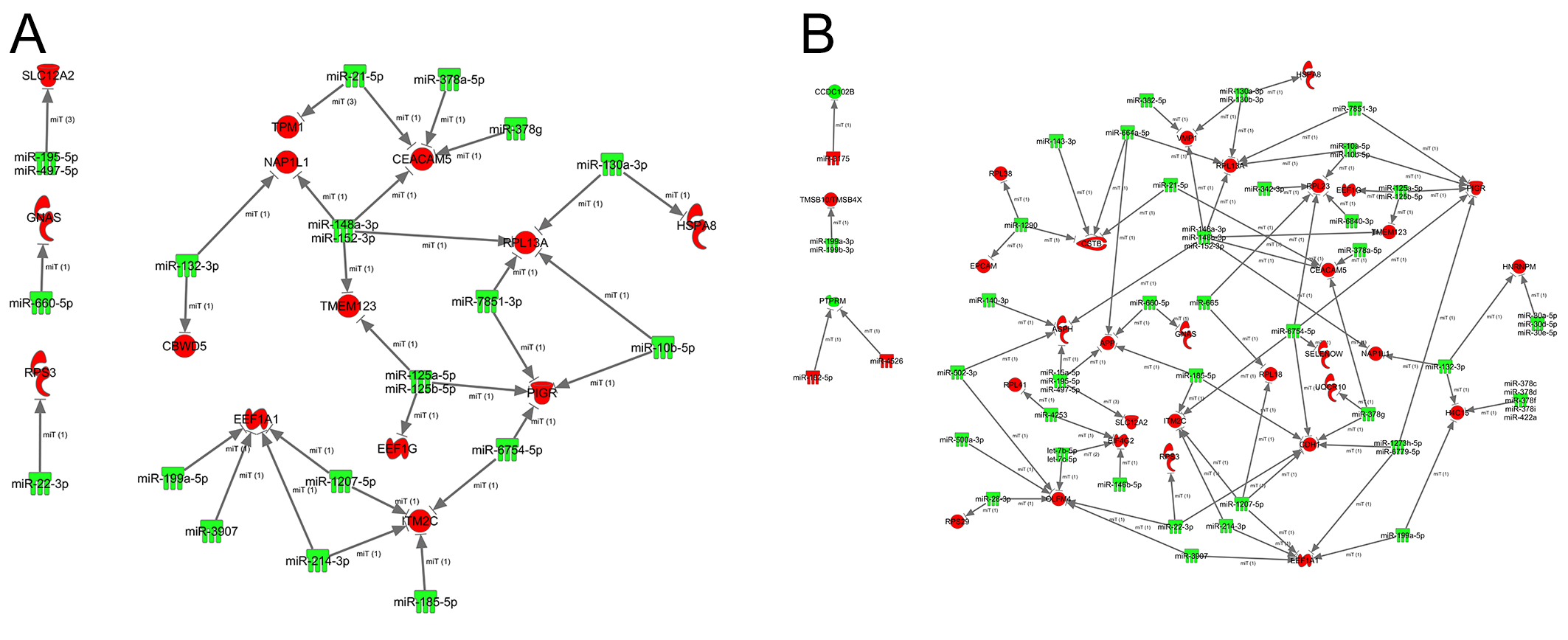

Supplement: Supplementary Figure 4 — Significant miRNA/mRNA pairs with inverse relationships in adenoma (A)components (A), IMC (B), and CRC with the MSS phenotype (C). [file Image_4.tif]
