# Supplementary material for: Genome-Wide Analysis of microRNA and mRNA Expression in Colorectal Intramucosal Neoplasia and Colorectal Cancer With a Microsatellite-Stable Phenotype Based on Adenoma-Carcinoma Sequences
Source: Front Oncol. 2022 Jul 7;12:831100. doi: 10.3389/fonc.2022.831100 (PMC9300861; doi:10.3389/fonc.2022.831100)
Supplement: Supplementary Figure 5 — miRNA/mRNA pairs with inverse relationships based on the adenoma–carcinoma sequence within the same tumor. (A) Adenoma and (B) IMC components. [file Presentation_1.pptx]

## Slide 1
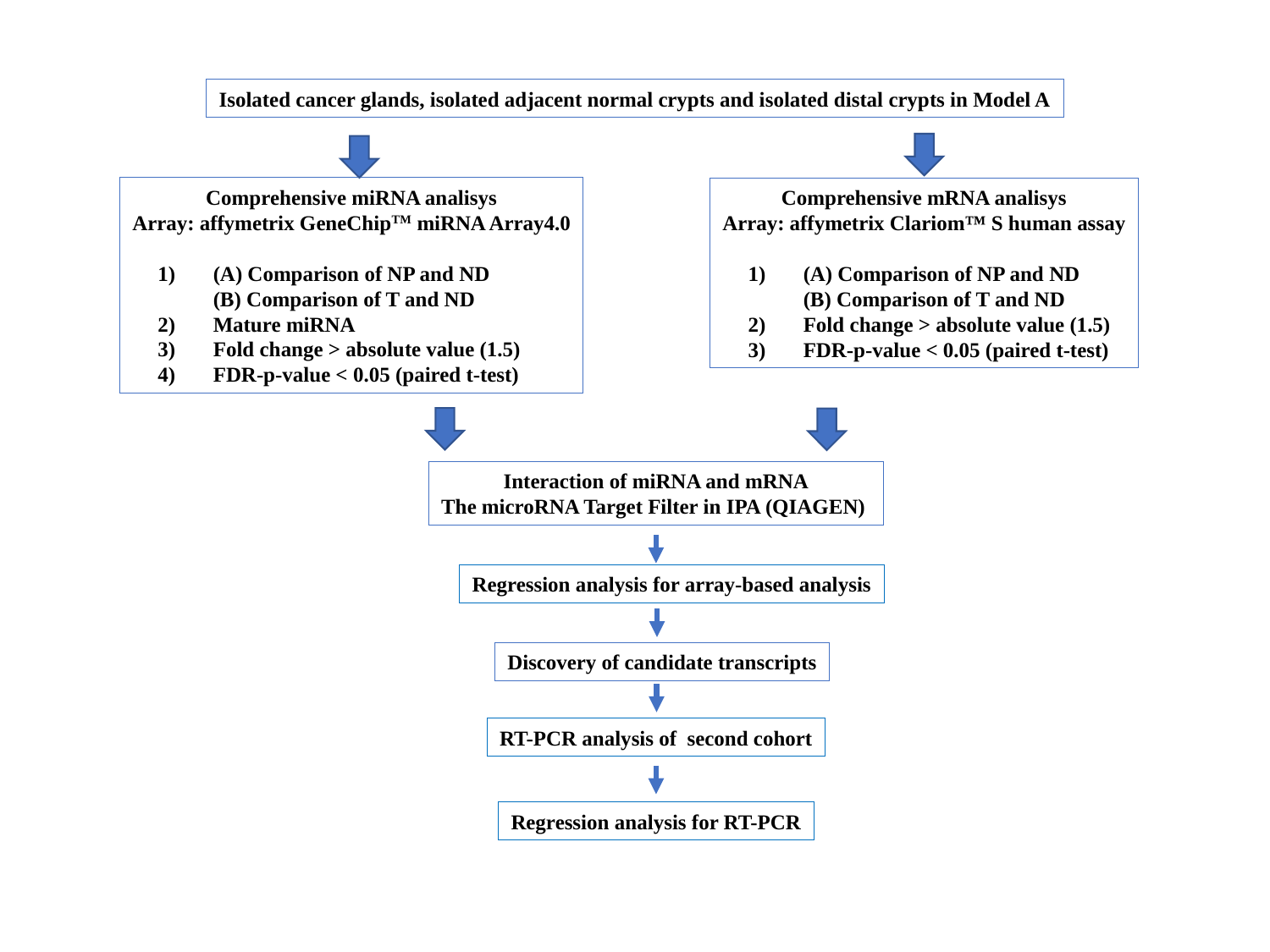

Isolated cancer glands, isolated adjacent normal crypts and isolated distal crypts in Model A
Comprehensive miRNA analisys
Array: affymetrix GeneChipTM miRNA Array4.0
1)	(A) Comparison of NP and ND
	(B) Comparison of T and ND
2)	Mature miRNA
3)	Fold change > absolute value (1.5)
4)	FDR-p-value < 0.05 (paired t-test)
Comprehensive mRNA analisys
Array: affymetrix Clariom™ S human assay
1)	(A) Comparison of NP and ND
	(B) Comparison of T and ND
2)	Fold change > absolute value (1.5)
3)	FDR-p-value < 0.05 (paired t-test)
Interaction of miRNA and mRNA
The microRNA Target Filter in IPA (QIAGEN)
Regression analysis for array-based analysis
Discovery of candidate transcripts
RT-PCR analysis of second cohort
Regression analysis for RT-PCR

## Slide 2
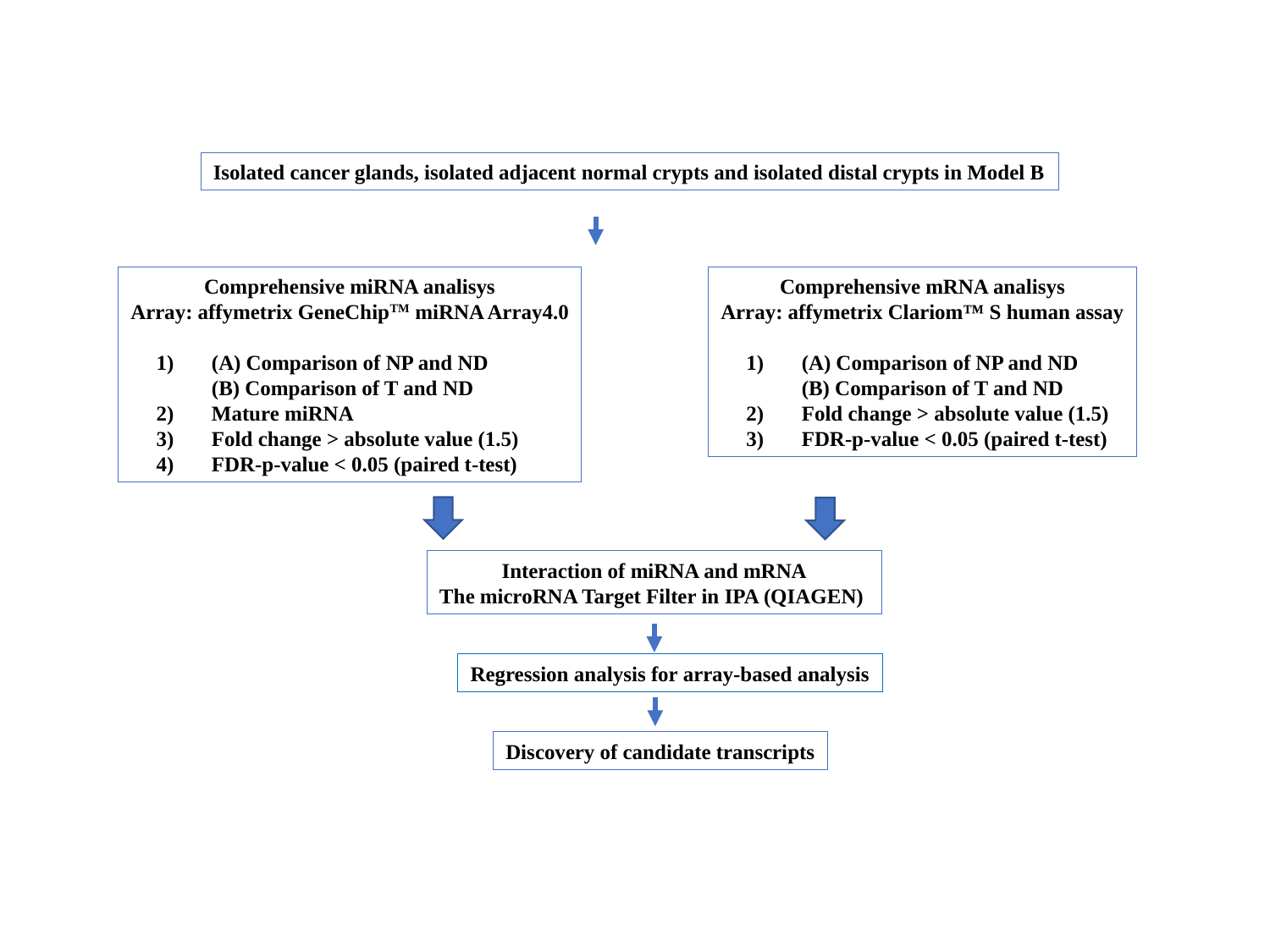

Isolated cancer glands, isolated adjacent normal crypts and isolated distal crypts in Model B
Comprehensive miRNA analisys
Array: affymetrix GeneChipTM miRNA Array4.0
1)	(A) Comparison of NP and ND
	(B) Comparison of T and ND
2)	Mature miRNA
3)	Fold change > absolute value (1.5)
4)	FDR-p-value < 0.05 (paired t-test)
Comprehensive mRNA analisys
Array: affymetrix Clariom™ S human assay
1)	(A) Comparison of NP and ND
	(B) Comparison of T and ND
2)	Fold change > absolute value (1.5)
3)	FDR-p-value < 0.05 (paired t-test)
Interaction of miRNA and mRNA
The microRNA Target Filter in IPA (QIAGEN)
Regression analysis for array-based analysis
Discovery of candidate transcripts
