# Supplementary material for: Genome-Wide Analysis of microRNA and mRNA Expression in Colorectal Intramucosal Neoplasia and Colorectal Cancer With a Microsatellite-Stable Phenotype Based on Adenoma-Carcinoma Sequences
Source: Front Oncol. 2022 Jul 7;12:831100. doi: 10.3389/fonc.2022.831100 (PMC9300861; doi:10.3389/fonc.2022.831100)
Supplement: Supplementary file 6 [file DataSheet_1.docx]

Supplementary Table 1. Primer lists we used in the present study

| **Gene Name** | **Asssay ID** |
| --- | --- |
| EEF1A1 | Hs05044986_g1 |
| GSKIP | Hs00212854_m1 |
| OLFM4 | Hs00197437_m1 |
| SH3BGRL3 | Hs00606772_g1 |
| SLC12A2 | Hs01074308_m1 |
| SLC26A3 | Hs00230798_m1 |
| ZG16 | Hs00380609_1 |
| miR-15a-5p | 389 |
| miR-15b-5p | 390 |
| miR-34a-5p | 426 |
| miR-195-5p | 494 |
| miR-502-3p | 2083 |
| miR-664b-3p | 479148_mir |
| miR-3064-5p | 465114_mat |
| miR-3907 | 465190_mat |
| miR-5093 | 480097_mir |
| miR-6807-5p | 466930_mat |

Supplementary Table 2-a. Simple regression analysis between micro RNA and mRNA in conventional adenoma using microarray (cohort 1).

| mRNA | micro RNA | Regression coefficient | Adjusted coefficient of determination | 95% CI | *p*-value |
| --- | --- | --- | --- | --- | --- |
| *SLC12A2* | hsa-miR-34a-5p | -0.767 | 0.371 | [-1.311, -0.223] | 0.0094 |
| *SLC12A2* | hsa-miR-15b-5p | -0.479 | 0.355 | [-0.831, -0.128] | 0.0113 |
| *SLC12A2* | hsa-miR-195-5p | -0.472 | 0.343 | [-0.825, -0.118] | 0.0128 |
| *EEF1A1* | hsa-miR-3907 | -1.035 | 0.334 | [-1.824, -0.246] | 0.0141 |
| *SLC12A2* | hsa-miR-15a-5p | -1.063 | 0.333 | [-1.876, -0.25] | 0.0143 |
| *SLC12A2* | hsa-miR-497-5p | -0.699 | 0.139 | [-1.536, 0.137] | 0.0942 |
| *ITM2C* | hsa-miR-214-3p | -0.888 | 0.113 | [-2.038, 0.262] | 0.1191 |
| *CSTB* | hsa-miR-143-3p | -0.339 | 0.059 | [-0.874, 0.196] | 0.1944 |
| *OLFM4* | hsa-let-7i-5p | -0.384 | 0.041 | [-1.042, 0.273] | 0.2290 |
| *PIGR* | hsa-miR-6776-5p | -1.172 | -0.002 | [-3.734, 1.39] | 0.3409 |
| *SLC12A2* | hsa-miR-421 | -0.494 | -0.010 | [-1.642, 0.653] | 0.3691 |
| *GNAS* | hsa-miR-34a-5p | -0.272 | -0.016 | [-0.937, 0.393] | 0.3927 |
| *ITM2C* | hsa-miR-1207-5p | -0.781 | -0.016 | [-2.693, 1.131] | 0.3933 |
| *OLFM4* | hsa-miR-28-3p | -0.397 | -0.026 | [-1.462, 0.667] | 0.4345 |
| *OLFM4* | hsa-let-7b-5p | -0.196 | -0.047 | [-0.89, 0.498] | 0.5522 |
| *EEF1A1* | hsa-miR-324-3p | -0.384 | -0.048 | [-1.766, 0.998] | 0.5586 |
| *PIGR* | hsa-miR-125b-5p | -0.331 | -0.048 | [-1.53, 0.868] | 0.5611 |
| *OLFM4* | hsa-miR-3907 | -0.213 | -0.059 | [-1.198, 0.773] | 0.6485 |
| *EEF1A1* | hsa-miR-214-3p | -0.184 | -0.061 | [-1.085, 0.717] | 0.6658 |
| *ITM2C* | hsa-miR-185-5p | -0.198 | -0.062 | [-1.188, 0.792] | 0.6729 |
| *OLFM4* | hsa-miR-500a-3p | -0.314 | -0.062 | [-1.929, 1.3] | 0.6809 |
| *OLFM4* | hsa-miR-151a-5p | -0.121 | -0.066 | [-0.841, 0.598] | 0.7218 |
| *OLFM4* | hsa-miR-502-3p | -0.229 | -0.067 | [-1.628, 1.17] | 0.7295 |
| *TMSB10* | hsa-miR-34a-5p | -0.152 | -0.070 | [-1.281, 0.977] | 0.7759 |
| *EEF1A1* | hsa-miR-1207-5p | -0.178 | -0.071 | [-1.584, 1.229] | 0.7893 |
| *TMSB10* | hsa-miR-199b-3p | -0.125 | -0.071 | [-1.129, 0.878] | 0.7918 |
| *ITM2C* | hsa-miR-4656 | -0.217 | -0.072 | [-2.156, 1.721] | 0.8124 |
| *PIGR* | hsa-miR-10b-5p | -0.207 | -0.072 | [-2.117, 1.703] | 0.8185 |
| *OLFM4* | hsa-miR-22-3p | -0.081 | -0.073 | [-0.852, 0.69] | 0.8239 |
| *GNAS* | hsa-miR-660-5p | -0.098 | -0.074 | [-1.238, 1.042] | 0.8559 |
| *CSTB* | hsa-miR-664a-5p | -0.036 | -0.076 | [-1.013, 0.941] | 0.9376 |
| *ITM2C* | hsa-miR-4270 | -0.051 | -0.077 | [-2.595, 2.493] | 0.9661 |
| *CEACAM5* | hsa-miR-378g | 0.565 |  |  |  |
| *CEACAM5* | hsa-miR-378a-5p | 1.208 |  |  |  |
| *CEACAM5* | hsa-miR-152-3p | 0.587 |  |  |  |
| *CEACAM5* | hsa-miR-21-5p | 0.172 |  |  |  |
| *CEACAM5* | hsa-miR-151a-3p | 0.337 |  |  |  |
| *SPTBN1* | hsa-miR-432-5p | 0.264 |  |  |  |
| *OLFM4* | hsa-miR-151b | 0.121 |  |  |  |
| *PIGR* | hsa-miR-1273h-5p | 1.078 |  |  |  |
| *PIGR* | hsa-miR-7851-3p | 0.639 |  |  |  |
| *PIGR* | hsa-miR-4270 | 0.480 |  |  |  |
| *PIGR* | hsa-miR-125a-5p | 0.111 |  |  |  |
| *PIGR* | hsa-miR-10a-5p | 0.006 |  |  |  |
| *TMSB10* | hsa-miR-152-3p | 0.155 |  |  |  |
| *TMSB10* | hsa-miR-200a-5p | 0.266 |  |  |  |
| *TMSB10* | hsa-miR-27b-3p | 0.003 |  |  |  |
| *TMSB10* | hsa-miR-1301-3p | 0.041 |  |  |  |
| *EEF1A1* | hsa-miR-151a-3p | 0.219 |  |  |  |
| *EEF1A1* | hsa-miR-1273h-5p | 1.450 |  |  |  |
| *EEF1A1* | hsa-miR-199a-5p | 1.086 |  |  |  |
| *CAST* | hsa-miR-19b-3p | 0.105 |  |  |  |
| *CSTB* | hsa-miR-21-5p | 0.039 |  |  |  |
| *CSTB* | hsa-miR-622 | 0.392 |  |  |  |
| *ITM2C* | hsa-miR-194-3p | 0.675 |  |  |  |
| *RPS21* | hsa-miR-193b-3p | 1.228 |  |  |  |
| *RPS21* | hsa-miR-425-5p | 0.152 |  |  |  |

CI, Confidence Interval.

Supplementary Table 2-b. Simple regression analysis between micro RNA and mRNA in intramucosal cancer using microarray (cohort 1).

| mRNA | micro RNA | Regression coefficient | Adjusted coefficient of determination | 95% CI | *p*-value |
| --- | --- | --- | --- | --- | --- |
| *GSKIP* | hsa-miR-664b-3p | -0.251 | 0.422 | [-0.499, -0.003] | 0.0483 |
| *OLFM4* | hsa-miR-502-3p | -0.457 | 0.419 | [-0.912, -0.003] | 0.0490 |
| *OLFM4* | hsa-let-7i-5p | -0.131 | -0.048 | [-0.521, 0.258] | 0.4405 |
| *OLFM4* | hsa-miR-28-3p | -0.107 | -0.084 | [-0.496, 0.281] | 0.5246 |
| *OLFM4* | hsa-miR-221-3p | -0.038 | -0.155 | [-0.408, 0.332] | 0.8102 |
| *OLFM4* | hsa-miR-151b | -0.025 | -0.165 | [-0.685, 0.635] | 0.9302 |
| *OLFM4* | hsa-miR-22-3p | 0.034 |  |  |  |
| *OLFM4* | hsa-miR-6752-5p | 0.033 |  |  |  |
| *FABP1* | hsa-miR-4505 | 0.854 |  |  |  |
| *FABP1* | hsa-miR-1275 | 0.342 |  |  |  |
| *TMX2* | hsa-miR-1275 | 0.346 |  |  |  |
| *TMX2* | hsa-miR-3175 | 0.075 |  |  |  |

CI, Confidence Interval.

Supplementary Table 2-c. Simple regression analysis between micro RNA and mRNA in colorectal cancer with an MSS phenotype using microarray (cohort 1).

| mRNA | micro RNA | Regression coefficient | Adjusted coefficient of determination | 95% CI | *p*-value |
| --- | --- | --- | --- | --- | --- |
| *SLC26A3* | hsa-miR-5093 | -1.365 | 0.385 | [-2.186, -0.543] | 0.0027 |
| *ZG16* | hsa-miR-6807-5p | -0.913 | 0.282 | [-1.591, -0.235] | 0.0113 |
| *SH3BGRL3* | hsa-miR-3064-5p | -0.463 | 0.236 | [-0.844, -0.082] | 0.0202 |
| *PIGR* | hsa-miR-6847-5p | -1.050 | 0.112 | [-2.274, 0.174] | 0.0880 |
| *SH3BGRL3* | hsa-miR-574-5p | -0.510 | 0.103 | [-1.125, 0.105] | 0.0984 |
| *FABP1* | hsa-miR-1306-3p | -1.433 | 0.088 | [-3.263, 0.397] | 0.1168 |
| *ITM2C* | hsa-miR-8071 | -0.576 | 0.068 | [-1.375, 0.223] | 0.1465 |
| *CNDP2* | hsa-miR-660-3p | -0.427 | 0.066 | [-1.025, 0.171] | 0.1507 |
| *CEACAM7* | hsa-miR-3127-5p | -0.867 | 0.047 | [-2.196, 0.462] | 0.1865 |
| *ITM2C* | hsa-miR-3127-5p | -0.657 | -0.034 | [-2.838, 1.524] | 0.5336 |
| *FABP1* | hsa-miR-660-3p | -0.192 | -0.056 | [-2.139, 1.754] | 0.8374 |
| *MS4A12* | hsa-miR-151a-3p | -0.065 | -0.056 | [-0.755, 0.624] | 0.8437 |
| *MS4A12* | hsa-miR-3148 | -0.064 | -0.057 | [-0.785, 0.657] | 0.8535 |
| *ODF1* | hsa-miR-22-3p | -0.001 | -0.059 | [-0.471, 0.469] | 0.9957 |
| *CEACAM7* | hsa-miR-663b | 0.656 |  |  |  |
| *CEACAM7* | hsa-miR-4646-5p | 0.521 |  |  |  |
| *ZG16* | hsa-miR-4646-5p | 0.519 |  |  |  |
| *B2M* | hsa-miR-595 | 0.025 |  |  |  |
| *B2M* | hsa-miR-130b-3p | 0.194 |  |  |  |
| *TSPAN1* | hsa-miR-151a-3p | 0.165 |  |  |  |
| *ITM2C* | hsa-miR-214-3p | 1.355 |  |  |  |
| *FABP1* | hsa-miR-130b-3p | 0.988 |  |  |  |
| *SH3BGRL3* | hsa-miR-214-3p | 0.671 |  |  |  |

MSS, microsatellite stable; CI, Confidence Interval.

Supplementary Table 3-a. Simple regression analysis between micro RNA and mRNA in adenoma component

| mRNA | micro RNA | Regression coefficient | Adjusted coefficient of determination | 95% CI | p-value |
| --- | --- | --- | --- | --- | --- |
| *RPS3* | hsa-miR-22-3p | -0.294 | 0.506 | [-0.456, -0.132] | 0.0018 |
| *HSPA8* | hsa-miR-130a-3p | -0.797 | 0.457 | [-1.279, -0.316] | 0.0034 |
| *CBWD5* | hsa-miR-132-3p | -0.427 | 0.398 | [-0.715, -0.139] | 0.007 |
| *PIGR* | hsa-miR-125b-5p | -0.488 | 0.335 | [-0.859, -0.116] | 0.014 |
|  | hsa-miR-125a-5p | -0.362 | 0.153 | [-0.78, 0.055] | 0.0833 |
|  | hsa-miR-10b-5p | -0.452 | 0.137 | [-0.995, 0.092] | 0.0957 |
|  | hsa-miR-6754-5p | -0.511 | 0.123 | [-1.152, 0.13] | 0.1086 |
|  | hsa-miR-7851-3p | -0.318 | -0.026 | [-1.174, 0.538] | 0.4365 |
| *NAP1L1* | hsa-miR-132-3p | -0.701 | 0.274 | [-1.305, -0.096] | 0.0264 |
|  | hsa-miR-148a-3p | -0.34 | 0.202 | [-0.685, 0.005] | 0.0528 |
|  | hsa-miR-152-3p | -0.192 | 0.038 | [-0.525, 0.141] | 0.2342 |
| *EEF1A1* | hsa-miR-3907 | -0.489 | 0.259 | [-0.924, -0.054] | 0.0303 |
|  | hsa-miR-199a-5p | -0.256 | 0.133 | [-0.568, 0.056] | 0.0994 |
|  | hsa-miR-214-3p | -0.2 | 0.055 | [-0.521, 0.121] | 0.2017 |
|  | hsa-miR-1207-5p | -0.272 | 0.007 | [-0.834, 0.289] | 0.3138 |
| *SLC12A2* | hsa-miR-497-5p | -0.507 | 0.187 | [-1.041, 0.027] | 0.0609 |
|  | hsa-miR-195-5p | -0.147 | 0.024 | [-0.421, 0.127] | 0.2668 |
| *TMEM123* | hsa-miR-148a-3p | -0.339 | 0.105 | [-0.789, 0.112] | 0.1282 |
|  | hsa-miR-125b-5p | -0.166 | 0.015 | [-0.491, 0.159] | 0.2895 |
|  | hsa-miR-125a-5p | -0.148 | -0.003 | [-0.475, 0.178] | 0.3439 |
|  | hsa-miR-152-3p | -0.162 | -0.023 | [-0.585, 0.261] | 0.4233 |
| *EEF1G* | hsa-miR-125a-5p | -0.139 | 0.079 | [-0.34, 0.063] | 0.1613 |
|  | hsa-miR-125b-5p | -0.139 | 0.079 | [-0.342, 0.063] | 0.1614 |
| *RPL13A* | hsa-miR-130a-3p | -0.189 | 0.066 | [-0.479, 0.101] | 0.1826 |
|  | hsa-miR-148a-3p | -0.161 | 0.065 | [-0.41, 0.087] | 0.1841 |
|  | hsa-miR-152-3p | -0.094 | -0.015 | [-0.321, 0.134] | 0.3897 |
|  | hsa-miR-10b-5p | -0.079 | -0.033 | [-0.31, 0.151] | 0.4699 |
|  | hsa-miR-7851-3p | 0.018 | -0.076 | [-0.322, 0.358] | 0.9118 |
| *GNAS* | hsa-miR-660-5p | -0.229 | 0.056 | [-0.595, 0.136] | 0.1984 |
| *CEACAM5* | hsa-miR-148a-3p | -0.294 | 0.048 | [-0.779, 0.191] | 0.2136 |
|  | hsa-miR-152-3p | -0.22 | 0.014 | [-0.654, 0.214] | 0.2935 |
|  | hsa-miR-21-5p | -0.111 | -0.047 | [-0.508, 0.285] | 0.5543 |
|  | hsa-miR-378a-5p | -0.14 | -0.06 | [-0.795, 0.516] | 0.6525 |
|  | hsa-miR-378g | -0.147 | -0.063 | [-0.922, 0.628] | 0.688 |
| *ITM2C* | hsa-miR-214-3p | -0.27 | -0.003 | [-0.867, 0.328] | 0.347 |
|  | hsa-miR-6754-5p | -0.277 | -0.005 | [-0.898, 0.344] | 0.3521 |
|  | hsa-miR-1207-5p | -0.349 | -0.035 | [-1.385, 0.687] | 0.4795 |
|  | hsa-miR-185-5p | -0.175 | -0.066 | [-1.197, 0.846] | 0.7164 |
| *TPM1* | hsa-miR-21-5p | 0.066 | -0.012 | [-0.089, 0.22] | 0.376 |

CI, Confidence Interval.

Supplementary Table 3-b. Simple regression analysis between micro RNA and mRNA in carcinoma component

| mRNA | micro RNA | Regression coefficient | Adjusted coefficient of determination | 95%CI | p-value |
| --- | --- | --- | --- | --- | --- |
| *APP* | hsa-miR-660-5p | -0.299 | 0.254 | [-0.567, -0.03] | 0.0319 |
|  | hsa-miR-664a-5p | -0.25 | 0.223 | [-0.492, -0.009] | 0.0433 |
|  | hsa-miR-195-5p | -0.122 | 0.15 | [-0.265, 0.02] | 0.0855 |
|  | hsa-miR-497-5p | -0.172 | 0.012 | [-0.516, 0.172] | 0.2999 |
|  | hsa-miR-15a-5p | -0.168 | 0 | [-0.529, 0.194] | 0.3345 |
|  | hsa-miR-185-5p | -0.091 | -0.048 | [-0.418, 0.236] | 0.5587 |
| *RPS3* | hsa-miR-22-3p | -0.229 | 0.198 | [-0.463, 0.005] | 0.0548 |
| *HSPA8* | hsa-miR-130a-3p | -0.609 | 0.198 | [-1.232, 0.015] | 0.0549 |
| *EEF1A1* | hsa-miR-1207-5p | -0.391 | 0.192 | [-0.797, 0.015] | 0.0576 |
|  | hsa-miR-3907 | -0.329 | 0.112 | [-0.756, 0.099] | 0.1206 |
|  | hsa-miR-1273h-5p | -0.166 | 0.013 | [-0.495, 0.163] | 0.2963 |
|  | hsa-miR-199a-5p | -0.132 | -0.031 | [-0.506, 0.242] | 0.4591 |
|  | hsa-miR-6779-5p | 0.163 | -0.045 | [-0.4, 0.726] | 0.5426 |
|  | hsa-miR-214-3p | -0.038 | -0.071 | [-0.341, 0.264] | 0.79 |
| *PIGR* | hsa-miR-6754-5p | -0.478 | 0.175 | [-0.997, 0.041] | 0.068 |
|  | hsa-miR-125b-5p | -0.404 | 0.11 | [-0.931, 0.124] | 0.1221 |
|  | hsa-miR-10b-5p | -0.562 | 0.106 | [-1.307, 0.183] | 0.1269 |
|  | hsa-miR-7851-3p | -0.46 | 0.041 | [-1.247, 0.328] | 0.2292 |
|  | hsa-miR-1273h-5p | -0.385 | 0.04 | [-1.046, 0.276] | 0.2304 |
|  | hsa-miR-125a-5p | -0.298 | 0.035 | [-0.821, 0.225] | 0.2406 |
|  | hsa-miR-10a-5p | -0.121 | -0.066 | [-0.846, 0.604] | 0.7246 |
|  | hsa-miR-6779-5p | 0.176 | -0.068 | [-0.983, 1.335] | 0.748 |
| *HIST2H4B* | hsa-miR-132-3p | -0.302 | 0.075 | [-0.749, 0.144] | 0.075154 |
|  | hsa-miR-199a-5p | -0.112 | 0.004 | [-0.349, 0.124] | 0.3223 |
|  | hsa-miR-378d | -0.116 | -0.026 | [-0.428, 0.197] | 0.4383 |
|  | hsa-miR-422a | -0.079 | -0.03 | [-0.301, 0.143] | 0.4558 |
|  | hsa-miR-378f | -0.061 | -0.034 | [-0.239, 0.118] | 0.4759 |
|  | hsa-miR-378c | -0.06 | -0.038 | [-0.245, 0.125] | 0.4962 |
|  | hsa-miR-378i | -0.029 | -0.07 | [-0.239, 0.181] | 0.7711 |
| *CDH1* | hsa-miR-1207-5p | -0.483 | 0.149 | [-1.045, 0.078] | 0.0858 |
|  | hsa-miR-6754-5p | -0.237 | 0.064 | [-0.603, 0.129] | 0.1853 |
|  | hsa-miR-185-5p | 0.368 | 0.044 | [-0.251, 0.987] | 0.2214 |
|  | hsa-miR-6779-5p | -0.387 | 0.02 | [-1.122, 0.348] | 0.2763 |
|  | hsa-miR-1273h-5p | -0.155 | -0.034 | [-0.61, 0.299] | 0.4733 |
|  | hsa-miR-22-3p | 0.043 | -0.073 | [-0.375, 0.462] | 0.8264 |
|  | hsa-miR-378g | -0.028 | -0.076 | [-0.676, 0.62] | 0.928 |
| *NAP1L1* | hsa-miR-132-3p | -0.597 | 0.143 | [-1.304, 0.11] | 0.0911 |
|  | hsa-miR-152-3p | -0.169 | 0.002 | [-0.53, 0.191] | 0.3287 |
|  | hsa-miR-148a-3p | -0.124 | -0.044 | [-0.054, 0.292] | 0.5312 |
|  | hsa-miR-148b-3p | -0.094 | -0.071 | [-0.861, 0.673] | 0.795 |
| *EEF1G* | hsa-miR-125a-5p | -0.182 | 0.11 | [-0.42, 0.056] | 0.1222 |
|  | hsa-miR-125b-5p | -0.178 | 0.085 | [-0.431, 0.076] | 0.1538 |
| *EIF4G2* | hsa-miR-4253 | -0.293 | 0.109 | [-0.677, 0.091] | 0.1231 |
|  | hsa-let-7b-5p | 0.372 | 0.063 | [-0.206, 0.949] | 0.1877 |
|  | hsa-let-7c-5p | 0.307 | 0.038 | [-0.226, 0.839] | 0.2357 |
|  | hsa-miR-146b-5p | 0.061 | -0.069 | [-0.37, 0.492] | 0.765 |
| *CCDC102B* | hsa-miR-3175 | -0.455 | 0.108 | [-1.052, 0.143] | 0.1243 |
| *OLFM4* | hsa-let-7c-5p | -0.473 | 0.099 | [-1.115, 0.169] | 0.1355 |
|  | hsa-miR-3907 | -0.303 | 0.023 | [-0.872, 0.266] | 0.2704 |
|  | hsa-miR-22-3p | -0.198 | 0.02 | [-0.575, 0.178] | 0.2761 |
|  | hsa-miR-502-3p | -0.292 | 0.019 | [-0.853, 0.268] | 0.2805 |
|  | hsa-miR-28-3p | -0.192 | 0.014 | [-0.569, 0.186] | 0.2934 |
|  | hsa-let-7b-5p | -0.336 | -0.003 | [-1.081, 0.408] | 0.3467 |
|  | hsa-miR-500a-3p | -0.156 | -0.041 | [-0.658, 0.345] | 0.5126 |
| *GNAS* | hsa-miR-660-5p | -0.406 | 0.096 | [-0.963, 0.15] | 0.1389 |
| *TMEM123* | hsa-miR-148b-3p | -0.718 | -1.727 | [0.29, 0.089] | 0.1479 |
|  | hsa-miR-125a-5p | 0.109 | -0.05 | [-0.295, 0.513] | 0.5716 |
|  | hsa-miR-152-3p | -0.086 | -0.067 | [-0.618, 0.446] | 0.7322 |
|  | hsa-miR-148a-3p | -0.038 | -0.075 | [-0.641, 0.566] | 0.8952 |
|  | hsa-miR-125b-5p | -0.004 | -0.077 | [-0.433, 0.426] | 0.9859 |
| *CSTB* | hsa-miR-664a-5p | -0.294 | 0.067 | [-0.742, 0.155] | 0.1805 |
|  | hsa-miR-143-3p | -0.147 | 0.043 | [-0.395, 0.101] | 0.2235 |
|  | hsa-miR-1290 | 0.028 | -0.075 | [-0.413, 0.468] | 0.8939 |
|  | hsa-miR-21-5p | 0.011 | -0.077 | [-0.359, 0.381] | 0.9492 |
| *RPL23* | hsa-miR-665 | -0.152 | 0.05 | [-0.402, 0.097] | 0.21 |
|  | hsa-miR-342-3p | -0.087 | -0.006 | [-0.284, 0.11] | 0.3561 |
|  | hsa-miR-6840-3p | -0.045 | -0.069 | [-0.353, 0.262] | 0.7546 |
| *VMP1* | hsa-miR-130a-3p | -0.21 | 0.05 | [-0.555, 0.135] | 0.2106 |
|  | hsa-miR-152-3p | -0.164 | 0.025 | [-0.467, 0.14] | 0.2645 |
|  | hsa-miR-148a-3p | -0.112 | -0.039 | [-0.465, 0.241] | 0.5041 |
|  | hsa-miR-382-5p | -0.104 | -0.056 | [-0.552, 0.344] | 0.6244 |
|  | hsa-miR-130b-3p | 0.015 | -0.076 | [-0.26, 0.291] | 0.9073 |
|  | hsa-miR-148b-3p | 0.023 | -0.076 | [-0.63, 0.677] | 0.9397 |
| *RPL18* | hsa-miR-665 | -0.139 | 0.045 | [-0.371, 0.094] | 0.2193 |
|  | hsa-miR-1207-5p | -0.157 | -0.009 | [-0.522, 0.207] | 0.3681 |
| *TMSB10* | hsa-miR-199a-3p | -0.166 | 0.041 | [-0.45, 0.117] | 0.2273 |
|  | hsa-miR-199b-3p | -0.166 | 0.041 | [-0.45, 0.117] | 0.2273 |
| *ASPH* | hsa-miR-140-3p | 0.264 | 0.031 | [-0.211, 0.738] | 0.2512 |
|  | hsa-miR-497-5p | -0.312 | -0.016 | [-1.078, 0.454] | 0.3944 |
|  | hsa-miR-148b-3p | -0.42 | -0.019 | [-1.477, 0.637] | 0.4059 |
|  | hsa-miR-15a-5p | -0.275 | -0.034 | [-1.081, 0.531] | 0.4742 |
|  | hsa-miR-502-3p | 0.191 | -0.047 | [-0.49, 0.873] | 0.5549 |
|  | hsa-miR-152-3p | -0.14 | -0.05 | [-0.663, 0.382] | 0.5716 |
|  | hsa-miR-148a-3p | -0.087 | -0.069 | [-0.692, 0.509] | 0.7576 |
|  | hsa-miR-195-5p | -0.017 | -0.076 | [-0.367, 0.334] | 0.9204 |
| *PTPRM* | hsa-miR-4526 | -0.188 | 0.03 | [-0.528, 0.152] | 0.2527 |
|  | hsa-miR-182-5p | 0.089 | -0.004 | [-0.109, 0.287] | 0.3483 |
| *HNRNPM* | hsa-miR-30a-5p | -0.173 | 0.019 | [-0.505, 0.159] | 0.2796 |
|  | hsa-miR-30d-5p | -0.101 | -0.041 | [-0.423, 0.222] | 0.5122 |
|  | hsa-miR-30e-5p | -0.148 | -0.048 | [-0.686, 0.39] | 0.5616 |
|  | hsa-miR-132-3p | -0.128 | -0.066 | [-0.874, 0.619] | 0.7181 |
| *RPL13A* | hsa-miR-130a-3p | -0.119 | 0.004 | [-0.368, 0.13] | 0.3215 |
|  | hsa-miR-664a-5p | -0.121 | 0.004 | [-0.376, 0.134] | 0.3234 |
|  | hsa-miR-152-3p | -0.084 | -0.023 | [-0.303, 0.135] | 0.4218 |
|  | hsa-miR-10b-5p | -0.079 | -0.04 | [-0.329, 0.171] | 0.5066 |
|  | hsa-miR-10a-5p | 0.05 | -0.058 | [-0.175, 0.274] | 0.6418 |
|  | hsa-miR-148b-3p | -0.083 | -0.064 | [-0.541, 0.375] | 0.7003 |
|  | hsa-miR-148a-3p | -0.021 | -0.074 | [-0.274, 0.232] | 0.8617 |
|  | hsa-miR-7851-3p | 0.013 | -0.076 | [-0.246, 0.273] | 0.9125 |
|  | hsa-miR-130b-3p | 0.007 | -0.076 | [-0.187, 0.202] | 0.9358 |
| *PRL23* | hsa-miR-6754-5p | -0.105 | -0.009 | [-0.349, 0.138] | 0.3671 |
|  | hsa-miR-10b-5p | -0.1 | -0.045 | [-0.442, 0.242] | 0.5378 |
|  | hsa-miR-10a-5p | 0.005 | -0.077 | [-0.305, 0.314] | 0.9745 |
| *ITM2C* | hsa-miR-6754-5p | -0.213 | -0.014 | [-0.724, 0.298] | 0.3849 |
|  | hsa-miR-1207-5p | -0.288 | -0.032 | [-1.119, 0.542] | 0.4665 |
|  | hsa-miR-214-3p | -0.14 | -0.052 | [-0.683, 0.403] | 0.5869 |
|  | hsa-miR-185-5p | -0.052 | -0.076 | [-0.934, 0.829] | 0.9 |
| *CEACAM5* | hsa-miR-152-3p | -0.274 | -0.016 | [-0.942, 0.394] | 0.392 |
|  | hsa-miR-148b-3p | -0.535 | -0.021 | [-1.909, 0.84] | 0.4158 |
|  | hsa-miR-21-5p | 0.137 | -0.058 | [-0.48, 0.754] | 0.6387 |
|  | hsa-miR-378a-5p | 0.16 | -0.063 | [-0.676, 0.997] | 0.686 |
|  | hsa-miR-148a-3p | -0.061 | -0.075 | [-0.837, 0.714] | 0.8669 |
|  | hsa-miR-378g | 0.029 | -0.077 | [-0.904, 0.962] | 0.9476 |
| *RPL38* | hsa-miR-1290 | -0.143 | -0.024 | [-0.522, 0.236] | 0.429 |
| *SLC12A2* | hsa-miR-497-5p | -0.271 | -0.026 | [-0.996, 0.454] | 0.434 |
|  | hsa-miR-15a-5p | -0.262 | -0.033 | [-1.022, 0.497] | 0.4689 |
|  | hsa-miR-195-5p | -0.005 | -0.077 | [-0.336, 0.326] | 0.9754 |
| *RPL41* | hsa-miR-4253 | -0.127 | -0.027 | [-0.472, 0.218] | 0.4415 |
| *SEPW1* | hsa-miR-6754-5p | -0.076 | -0.056 | [-0.403, 0.251] | 0.6226 |
| *RPS29* | hsa-miR-28-3p | 0.052 | -0.064 | [-0.23, 0.338] | 0.6985 |
| *HSPA8* | hsa-miR-130b-3p | 0.054 | -0.073 | [-0.487, 0.596] | 0.8314 |
| *EPCAM* | hsa-miR-1290 | 0.038 | -0.074 | [-0.438, 0.514] | 0.8645 |
| *UQCR10* | hsa-miR-378g | 0.02 | -0.076 | [-0.309, 0.349] | 0.8978 |

CI, Confidence Interval.
